# Wild deer (*Pudu* *puda*) from Chile harbor a novel ecotype of *Anaplasma* *phagocytophilum*

**DOI:** 10.1186/s13071-023-05657-9

**Published:** 2023-01-27

**Authors:** Adriana Santodomingo, Richard Thomas, Sofía Robbiano, Juan E. Uribe, Catalina Parragué-Migone, Javier Cabello-Stom, Frank Vera-Otarola, Carola Valencia-Soto, Darío Moreira-Arce, Ezequiel Hidalgo-Hermoso, Sebastián Muñoz-Leal

**Affiliations:** 1 Departamento de Ciencia Animal, Facultad de Ciencias Veterinarias, Universidad de Concepción, Chillán, Chile; 2 Department of Biodiversity and Evolutionary Biology, Museo Nacional de Ciencias Naturales (MNCN-CSIC), 28006 Madrid, Spain; 3 Department of Invertebrate Zoology, National Museum of Natural History, Smithsonian Institution, Washington, DC 20013 USA; 4 Centro de Conservación de la Biodiversidad, Chiloé Silvestre, Nal Bajo, Chiloé, Chile; 5 Facultad de Ciencias de la Naturaleza, Sede de La Patagonia, Universidad San Sebastián, Puerto Montt, Chile; 6 Universidad de Santiago de Chile (USACH), Santiago, Chile; 7 Institute of Ecology and Biodiversity (IEB), Santiago, Chile; 8 Fundación Buin Zoo, Panamericana Sur Km 32, Buin, Chile

**Keywords:** Southern Pudu, *Ixodesstilesi*, Wildlife, Molecular detection, Phylogenetics, Tick-borne diseases

## Abstract

**Background:**

Deer species play an important role in the enzootic cycles of several *Anaplasma* species. While in the Northern Hemisphere ticks of genus *Ixodes* are well recognized vectors of these intracellular bacteria, less is known regarding the biological cycles of *Anaplasma* spp. in South America.

**Methods:**

Using PCR protocols and Sanger sequencing, we assessed the presence of *Anaplasma* spp. in blood and ticks collected on a native deer species (*Pudu* *puda*) from southern Chile.

**Results:**

Based on phylogenetic analyses of the 16S rRNA, *gltA* and *groEL* genes and calculation of average sequence divergence for *groEL*, our results bring to light a novel genovariant of *Anaplasma* *phagocytophilum* (named strain “Patagonia”). The strain represents a novel ecotype within the *A*. *phagocytophilum* species complex and was detected in both *P.* *puda* and their ticks. Using a larger matrix, denser taxon sampling and outgroup, our maximum-likelihood- and Bayesian-inferred phylogenies for *groEL* provide an accurate picture of the topology of *A*. *phagocytophilum* ecotypes and their evolutionary relationships.

**Conclusions:**

This is the first report of an ecotype of *A*. *phagocytophilum* in South America. Our results provide novel insight into the genetic diversity and ecology of this complex of bacterial lineages. Further studies should elucidate the enzootic cycle of *A*. *phagocytophilum* strain “Patagonia” and assess its pathogenic potential for pudues, domestic animals and humans in the region.

**Graphical Abstract:**

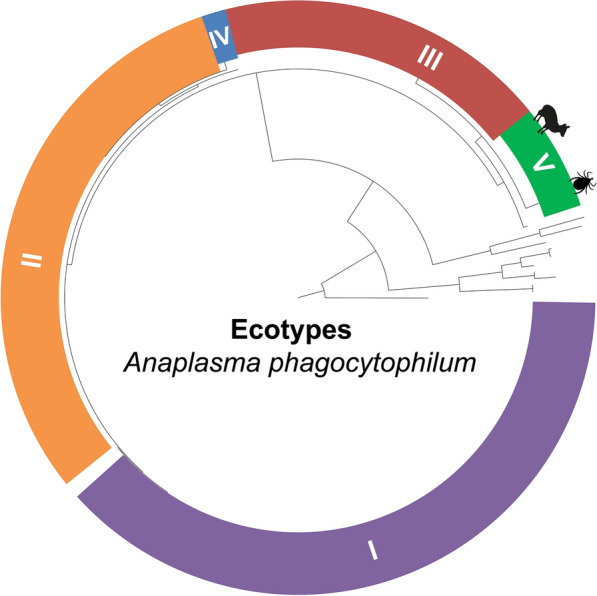

**Supplementary Information:**

The online version contains supplementary material available at 10.1186/s13071-023-05657-9.

## Background

Alphaproteobacteria in the genus *Anaplasma* are intracellular cocobacilli of mammal blood cells transmitted by ticks of genera *Amblyomma*, *Dermacentor*, *Hyalomma*, *Ixodes* and *Rhipicephalus* [1]. *Anaplasma* spp. are infectious agents that cause diseases ranging from harmless to fatal [2, 3]. Among five species and numerous genovariants that have been identified [1], *Anaplasma* *phagocytophilum* is of animal and public health relevance because of tick-borne fever in ruminants and granulocytic anaplasmosis in equines, canids, felids and humans in the Northern Hemisphere [4, 5].

The genetic diversity of *Anaplasma* spp. has been explored using the conserved 16S rRNA (*rrs*) gene [1]; however, due to its weak intraspecific discriminatory resolution [6], variable loci such as citrate synthase (*gltA*) and the heat-shock operon (*groEL*) have been selected as suitable markers for single-locus genetic analyses [1, 7, 8]. Based on these markers four ecotypes split into seven phylogenetic clusters have been proposed to compose the *A*. *phagocytophilum* complex in Europe, Asia and North America [1, 7, 8]. A bacterial ecotype is a monophyletic array of strains sharing a similar ecological niche [9, 10], for which the average sequence divergence among groups is significantly higher than the divergence within them for a given gene [9]. *Anaplasma* *phagocytophilum* ecotypes and clusters have been defined according to their genetics, geographic distribution, enzootic cycles, host preference and pathogenicity [7, 11]. For example, ticks of genus *Ixodes* and cervids constitute the ecological niche for *A.* *phagocytophilum* ecotypes I and II [1].

Cervids are reservoirs for *Anaplasma* spp. and are often parasitized by ticks of the genus *Ixodes* that transmit these bacteria [12]. For instance, in the Northern Hemisphere, *Ixodes* *scapularis* and *Ixodes* *pacificus* (USA), *Ixodes* *ricinus* (Europe), and *Ixodes* *persulcatus* (Eurasia) [13] are the known vectors of *A*. *phagocytophilum*. However, data on the epidemiology of *Anaplasma* spp. is vague in South American cervids [14–20], and restricted to few species from Brazil [14–17], Argentina [19] and Uruguay [18]. In Chile, temperate rainforests (roughly between 35º and 46º S) are the habitat for the pudu (*Pudu* *puda*), a deer species classified as near threatened [21], which is an important host of adults of the ticks *Ixodes* *stilesi* and *Ixodes* *taglei* [22]. Although the eco-epidemiological settings (i.e. *Ixodes* ticks and deer) for an ecotype of *A.* *phagocytophilum* to occur do exist in Chile, it is currently unknown whether the bacterium occupies this ecological niche in the country. In the present study, we analyzed blood and ticks collected directly from free-ranging pudues from southern Chile. Because only a few *Anaplasma* surveys performed in South American wild cervids have provided short sequences for the 16S rRNA locus (*rrs*) [14–19, 23], we performed genetic screenings with additional molecular markers to detect *Anaplasma* DNA to clarify inter- or intraspecific relationships.

## Methods

### Sample collection

**Fig. 1:**
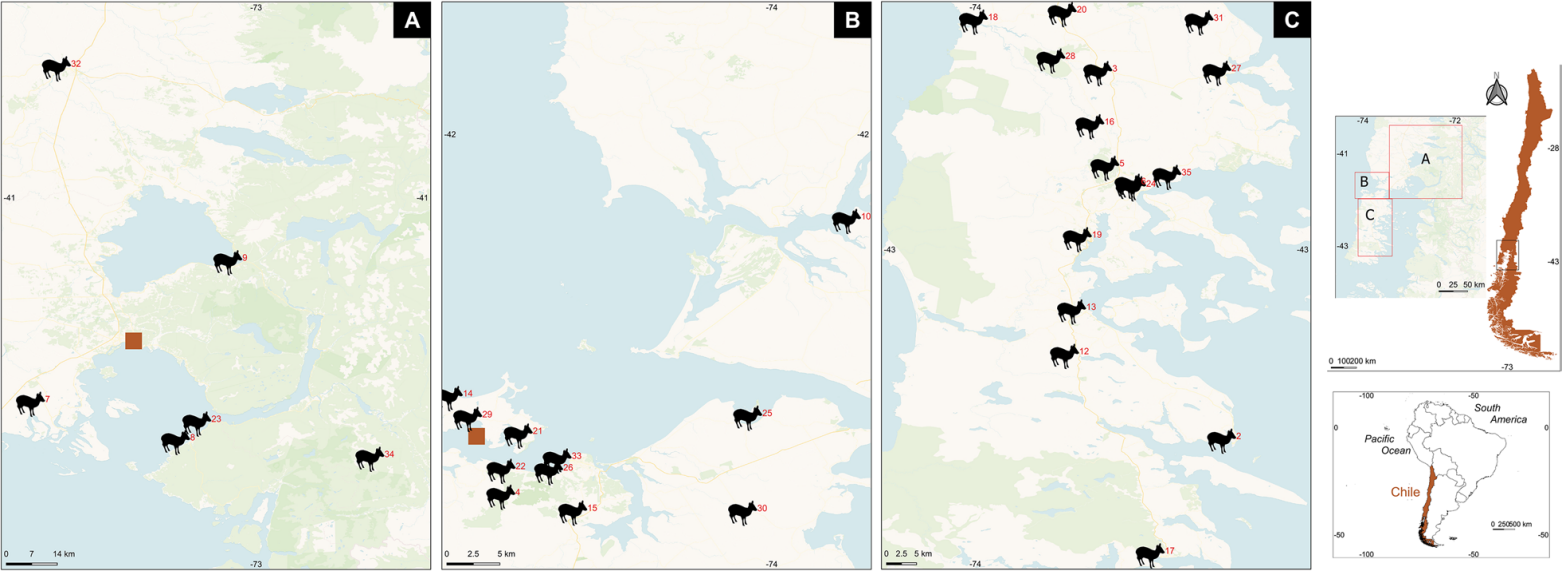
Map of Chile showing the origin of rescued pudues (black icons) within the Región de Los Lagos, Chile. Brown squares indicate the rehabilitation centers. Maps were constructed with QGIS 3.18.1-Zürich (https://www.gnu.org/licenses). QGIS, Quantum Geographic Information System

During a 5-year period (2017–2022), the blood (2–4 ml) of pudues admitted to any one of two wildlife rescue centers, Centro de Conservación Chiloé Silvestre (Nal Bajo, in Chiloé Island; − 41.839786, − 73.936015° W) and Cerefas Universidad San Sebastián (Puerto Montt; − 41.469628, − 72.907159), was collected from the cephalic or saphenous vein using an evacuated tube system (Vacutainer; Beckon, Dickson, and Company, Franklin Lakes, NJ, USA) on the day of admission (Fig. 1).

In addition to blood sampling, ticks were also removed with steel tweezers from various pudues. Blood samples and ectoparasites were kept in sterile tubes containing absolute ethanol and stored at − 80 °C until processing. The morphology of ticks was examined with a NexiusZoom (EVO) Stereo Microscope (Euromex Microscopen B.V., Arnhem, The Netherlands) and identified according to Nava et al. [22]. The identity of *Anaplasma*-positive ticks was further validated by sequencing a fragment of the tick mitochondrial (mt) 16S ribosomal RNA (rRNA) gene [22].

### DNA isolation

Genomic DNA was extracted with the DNeasy Blood & Tissue Kit (Qiagen, Hilden, Germany) according to the manufacturer’s protocol and eluted in 40 μl of buffer AE (10 mM Tris–Cl; 0.5 mM ethylenediaminetetraacetic acid [EDTA], pH 9.0). DNA was quantified with an Epoch™ Microplate Spectrophotometer (BioTek Instruments, Inc., Winooski, VT, USA) and assessed for quality at A260/A280 according to Khare et al. [24].

### Gene amplification and sequencing

The suitability of the extracted DNA was checked by a conventional PCR (cPCR) assay targeting the mammalian glyceraldehyde-3-phosphate dehydrogenase (*GAPDH*) and the tick mt 16S rRNA genes, respectively. The primers and thermal conditions used in this study together with their references are shown in Table 1. *Anaplasma* detection was achieved by implementing different nested and hemi-nested PCR protocols targeting the *rrs*, *gltA* and *groEL* genes. DNA of *Anaplasma* *platys* (OQ155255) was used as the positive control and nuclease-free water was used as the negative control. All PCR reactions were performed in a thermal cycler (ProFlexTM Base 32 × 3; Applied Biosystems, Thermo Fisher Scientific, Waltham, MA, USA) in a final reaction volume of 25 μl (12.5 μl DreamTaq Green PCR Master Mix [Thermo Fisher Scientific], 1 μl of each primer (0.4 μM), 8.5 μl of ultra-pure water and 2 μl template DNA. The PCR products were stained with GelRed® (Biotum, Tehran, Iran), separated by electrophoresis in 2% agarose gels and then visualized using an ENDURO™ GDS UV transilluminator (Labnet International, Edison, NJ, USA). Amplicons with bands of the expected size were purified and Sanger-sequenced at Macrogen (Seoul, South Korea).

**Table 1:** Primers and thermal conditions used for PCR detection and genetic characterization of *Anaplasma* and ticks

Organisms	Gene	PCR	Primer	Sequence	*T*_o_ (°C)	Expected length (in bp)	Reference
Mammals	*GAPDH*	Conventional	gapdh F	CCTTCATTGACCTCAACTACAT	52	400	[25]
			gapdh R	CCAAAGTTGTCATGGATGACC			
Ticks	Mitochondrial 16S rRNA (*rrs*)	Conventional	16S + 1	CCGGTCTCAACTCAGATCAAGT		460	[26]
			16S − 1	GCTCAATGATTTTTTAAATTGCTGT			
*Anaplasma*	16S rRNA (*rrs*)	Conventional	EC9	TACCTTGTTACGACTT	48	1300	[27]
			EC12A	TGATCCTGGCTCAGAACGAACG			[28]
		Nested	A17a	GCGGCAAGCCTCCCACAT	54	1200	[29]
			IS58-1345r	CACCAGCTTCGAGTTAAACC			
	*groEl*	Conventional	HS1a	AYTGGGCTGGTAYTGAAAT	47	1614	[30]
			GroEl_2R	CGTTCTTACTAGGAACATCAAC			[31]
		Nested	Gro677F	ATTACTCAGAGTGCTTCTCARTG	53	942	[32]
			GroEL_rev2	GCCGACTTTTAGTACAGCAA			[33]
		Heminested	GroEl_2F	TGTAAAGGCGCCTGGTTTCG	55	772	[31]
			GroEl_2R	CGTTCTTACTAGGAACATCAAC			[31]
	*gltA*	Conventional	F4b	CCAGGCTTTATGTCAACTGC	55	800	[34]
			R1b	CGATGACCAAAACCCAT			
		Nested	EHR-CS136F	TTYATGTCYACTGCTGCKTG	55	650	
			EHR-CS778R	GCNCCMCCATGMGCTGG			

*GAPDH* Glyceraldehyde-3-phosphate dehydrogenase, *gltA* citrate synthase gene, *groEL* heat-shock operon

### Assembly and sequence analyses

Amplicon sequences were quality-checked and edited with Geneious Prime® version (v) 2021.2.2 (www.geneious.com) to generate consensus sequences. Base calls with Phred values ≥ 20 were considered suitable for the analyses [35, 36]. The BLAST® tool (https://blast.ncbi.nlm.nih.gov) was employed to compare obtained nucleotide sequences and identify orthologous sequences.

### Phylogenetic analyses

Orthologous sequences downloaded from GenBank (https://www.ncbi.nlm.nih.gov) and consensus sequences were used to build alignments with the MAFFT multiple sequence alignment program using default parameters [37]. The alignments were subsequently trimmed and filtered with Block Mapping and Gathering with Entropy (BMGE) using default parameters to map informative regions for phylogenetics inferences [38].

Phylogenetic trees were constructed with the Bayesian inference (BI [39, 40]) and maximum-likelihood (ML [41]) methods in MrBayes v 3.2.6 [42] and IQ-TREE v 1.6.12 [43], respectively. As protein-coding genes present different nucleotide exchange rates (heterogeneity) at the first, second and third codon positions [42, 44], datasets were partitioned into the three codon positions (position-1, position-2 and position-3) [42, 44–46]. Then, the Model Finder command “TESTNEWONLYMERGE -mrate G” was implemented to select the best-fit evolutionary models and best-partition scheme for protein-coding gene datasets [47]. The ML best evolutionary models for non-coding genes were calculated using the ModelFinder command “-m TESTNEWONLY -mrate G” [47]. We used rapid hill-climbing and stochastic disturbance methods with 1000 ultrafast bootstrapping pseudo-replicates to evaluate the inferred tree robustness. Bootstrap values < 70%, 70–94% and ≥ 95% were considered non-significant, medium and solid statistical support [48], respectively.

BI phylogenies were constructed based on nucleotide substitution models selected with the MrBayes command "lset nst = mixed rates = gamma" for the non-coding dataset [42, 49]. On the other hand, the best partition schemes computed by ModelFinder and the MrBayes command “lset = mixed rates = invgamma” were used to calculate the best models for protein-encoding datasets [42, 46, 49]. Two independent tests of 20 × 10^6^ generations and four Markov chain Monte Carlo (MCMC) chains were implemented, sampling trees every 1000 generations and removing the first 25% as burn-in. Tracer v1.7.1 [50] was used to confirm the correlation and effective sample size of the MCMC. Bayesian posterior probabilities (BPP) with values > 0.70 in nodes were considered to indicate strong statistical support [51]. All best-fit models and partitions schemes were selected under the Bayesian Information Criterion (BIC) [52]. Trees were visualized and edited with FigTree v 1.4.1 (http://tree.bio.ed.ac.uk/software/figtree/) and Inkscape v 1.1 (https://inkscape.org/es/). Congruent topologies between ML and BI analyses were used to produce strict consensus trees in Geneious Prime with the Consensus Tree Builder tool, implementing a support threshold of 100%. The consensus phylogram included all monophyletic clades after comparing ML and BI topologies for each dataset.

### Genetic distance analyses

To assess the corrected pairwise distance and determine the average sequence divergence within and among ecotypes, an alignment of 936 bp was constructed with default parameters in MAFFT, including 214 *groEL* sequences of *A*. *phagocytophilum* with > 70% coverage between them, using *Anaplasma* *odocoilei* and *A*. *platys* as outgroups. The corrected pairwise distance was assessed using raxmlGUI [53, 54] for RAxML v 8 [55] with the GTR + GAMMA + I substitution model.

## Results

### Tick identification and blood samples

A total of 26 hard ticks and 55 blood samples were collected from pudues. All ticks were morphologically identified as *I.* *stilesi* (17 females, 5 males, 4 nymphs). Amplicons of the expected size were obtained for the mt 16S rRNA gene by PCR in 20 of the 26 tick specimens, with negative results obtained for six ticks (4 females, 1 male, 1 nymph), which were subsequently excluded from the analysis. PCR targeting the *GAPDH* gene in pudu blood resulted in amplicons of the expected size, confirming successful DNA extractions in all cases (Table 2).

**Table 2:** Sampled and *Anaplasma*-positive animals with the geographical coordinates of provenance

Species	Provenance	Locality^a^	Geographical coordinates (latitude, longitude)^b^	*Anaplasma* *phagocytophilum*^c^
*Pudu* *puda*	Continent	Cerefas Universidad San Sebastián, Puerto Montt (1)	− 41.469628, − 72.907159	1/3
	Island	Queilén (2)	− 42.885721, − 73.468359	0/3
	Island	Degañ (3)	− 42.145274, − 73.720717	0/1
	Island	Pauldeo (4)	− 41.908360, − 73.891784	0/2
	Island	Mocopulli (5)	− 42.336344, − 73.706289	0/2
	Island	Tehuaco (6)	− 42.372438, − 73.657162	0/1
	Continent	Calbuco (7)	− 41.677865, − 73.201237	0/1
	Continent	Contao (8)	− 41.803322, − 72.719169	0/2
	Continent	Ensenada (9)	− 41.213838, − 72.545666	0/1
	Continent	Peñol Bajo (10)	− 41.598174, − 73.498427	0/1
	Island	Centro de Conservación Chiloé Silvestre (11)	− 41.839786, − 73.936015	0/1
	Island	Lago Tarahuín (12)	− 42.714684, − 73.788520	0/1
	Island	Chonchi (13)	− 42.625050, − 73.774028	0/3
	Island	Chauman (14)	− 41.797195, − 73.951494	0/2
	Island	Mechaico (15)	− 41.926147, − 73.809907	1/1
	Island	Butalcura (16)	− 42.252443, − 73.736915	1/1
	Island	Quellón (17)	− 43.116902, − 73.613887	0/4
	Island	Chepu (18)	− 42.041574, − 73.973976	1/1
	Island	Castro (19)	− 42.480140, − 73.762413	0/3
	Island	Quichitúe (20)	− 42.026219, − 73.793279	0/1
	Island	Guapilacuy (21)	− 41.839390, − 73.871975	0/1
	Island	Lechagua (22)	− 41.879088, − 73.891482	0/1
	Continent	Caleta Puelche (23)	− 41.742766, − 72.648612	0/1
	Island	Dalcahue (24)	− 42.377552, − 73.651920	1/3
	Island	Caulin (25)	− 41.819313, − 73.610747	0/1
	Island	Hueihue (26)	− 41.880609, − 73.837354	0/1
	Island	Quemchi (27)	− 42.144713, − 73.478056	0/2
	Island	Puntra (28)	− 42.119891, − 73.816342	1/1
	Island	Sector Naval—Faro Corona (29)	− 41.820747, − 73.929428	0/2
	Island	El Quilar (30)	− 41.926431, − 73.616454	0/1
	Island	Lliuco (31)	− 42.042983, − 73.514278	0/2
	Continent	Osorno (32)	− 40.576192, − 73.114948	0/1
	Island	Ancud (33)	− 41.867489, − 73.827690	0/1
	Continent	Los Lagos (34)	− 41.858622, − 72.073451	0/1
	Island	Quiquel (35)	− 42.354553, − 73.579767	0/1
	*Total no. positive specimens*			*6/55*
*Ixodes* *stilesi*	Island	Centro de Conservación Chiloé Silvestre (11)	− 41.839786, − 73.936015	*8/20*

^a^Numbers in parentheses correlate to the sites shown in Fig. 1

^b^Presented in pure numeric format

^c^Number of positive specimens/number of specimens tested

### *Anaplasma* detection

*Anaplasma* DNA was amplified in 8/26 (30.8%) *I*. *stilesi* (1 nymph, 1 male, 6 females) and in 6/55 (10.9%) pudues (Table 2). Eleven identical sequences were obtained for *rrs* (1,212 bp), 12 for *gltA* (722 bp) and 13 for *groEL* (1,286 bp). Pairwise comparisons between generated sequences indicated one genotype for *rrs*, seven genotypes for *gltA* and 11 genotypes for *groEL*. A mitochondrial genotype of 429 bp retrieved for *Anaplasma*-positive ticks (OP750053) was 99.5% (428/430 bp, 100% query cover, 2 gaps, 0 E-value) identical with a previous sequence of *I*. *stilesi* from Chile (DQ061292) [56].

After BLASTn comparisons, the *rrs* genotype matched with 94.8% identity *A*. *phagocytophilum* isolate D2_2 (MK814406), detected in *Canis* *lupus* *familiaris* from South Africa [57]; the *gltA* genotypes showed an identity ranging from 82.9% to 83.1% with *A*. *phagocytophilum* strain Sheep (KP861639) detected in an *Ixodes* sp. collected on a Norwegian White Sheep [58]; and the *groEL* genotypes were 91.4–91.8% identical with *A*. *phagocytophilum* samc001 (LC496077) detected in *Canis* *lupus* *familiaris* from Japan [59].

**Fig. 2:**
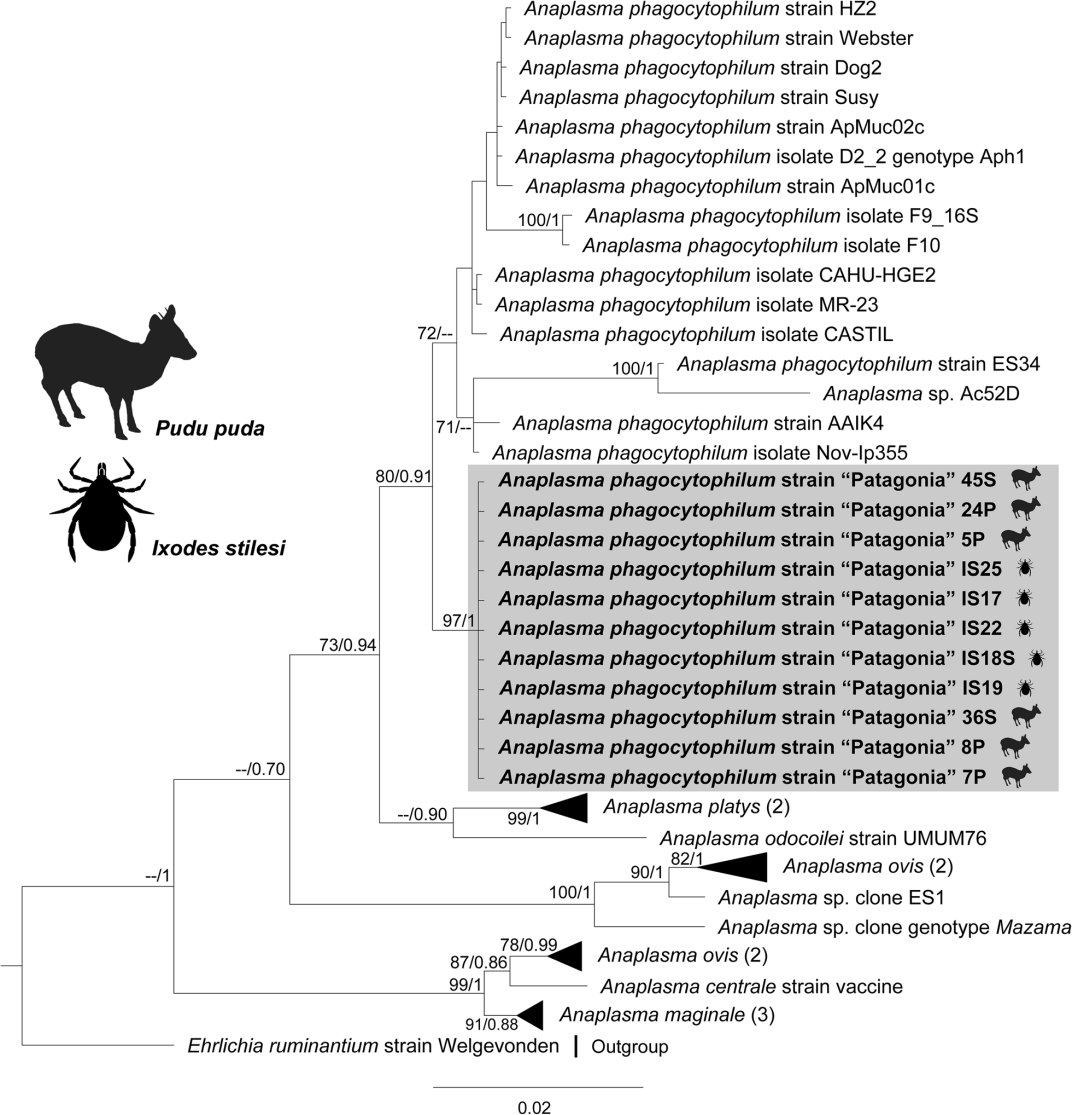
Maximum likelihood (ML) and Bayesian inference (BI) *rrs* gene consensus tree inferred for a subset of *Anaplasma* spp., using 41 sequences and an alignment of 1,382 bp. Best-fit evolutionary models calculated for the ML and BI methods were TPM3u + F + G4; and *M*_90_, *M*_177_, *M*_85_, *M*_152_, *M*_179_, *M*_117_, *M*_195_, respectively. Bootstrap values and Bayesian posterior probabilities (BPP) are indicated above or below each branch. The position of the strain of *Anaplasma* *phagocytophilum* characterized in the present study is highlighted in a gray box

**Fig. 3:**
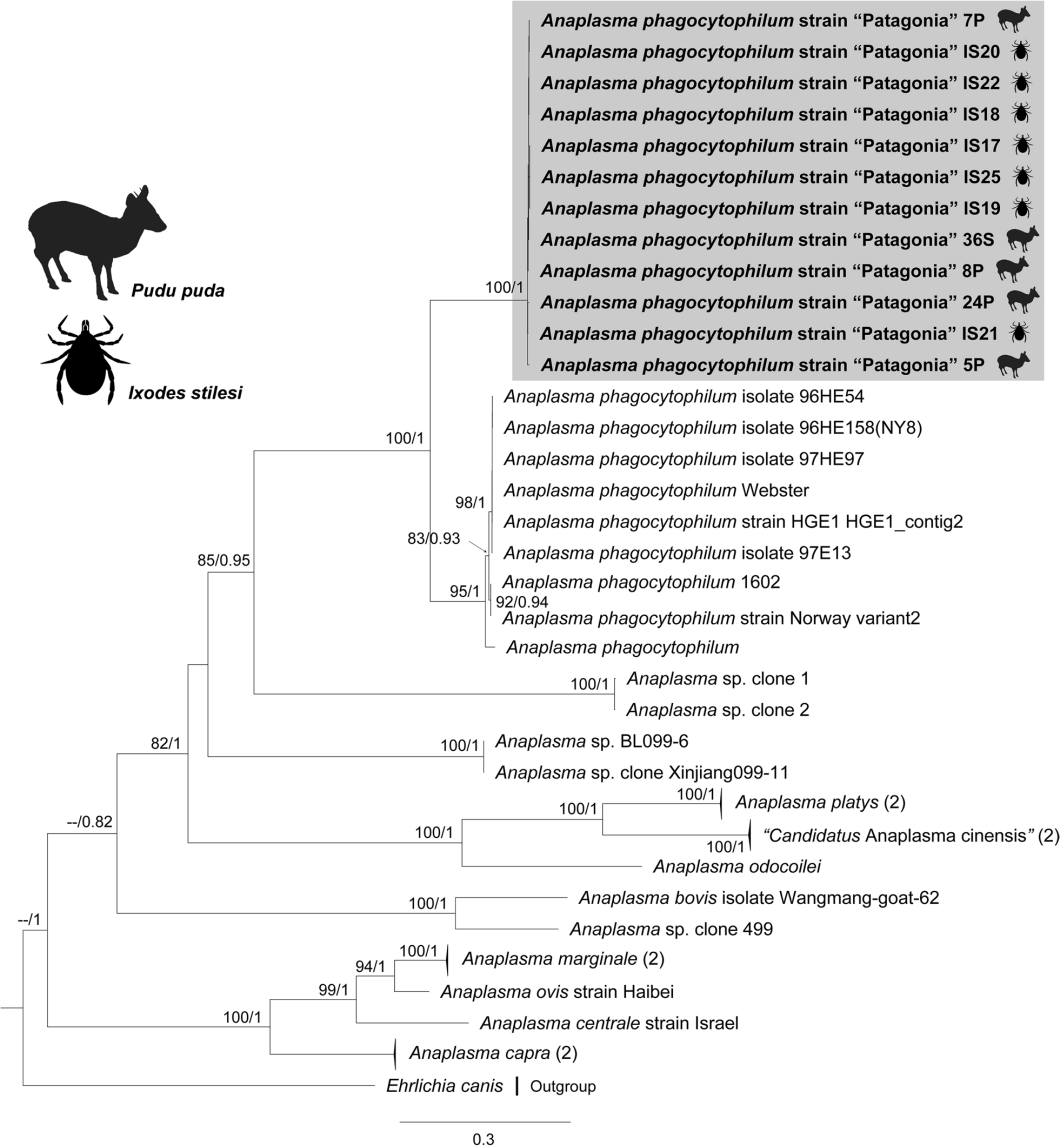
ML and BI consensus tree inferred for a subset of *Anaplasma* spp., using 40 sequences of the *gltA* gene and an alignment of 1152 bp. Best-fit evolutionary models calculated for the ML and BI methods were GTR + F + I + G4 (position-1), GTR + F + G4 (position-2), HKY + F + I + G4 (position-3); and *M*_64_, *M*_175_, *M*_173_, *M*_25_, *M*_171_, *M*_50_, *M*_125_ (position-1); *M*_80_, *M*_135_, *M*_164_, *M*_166_, *M*_145_ (position-2); *M*_90_, *M*_177_, *M*_152_, *M*_183_, *M*_136_ (position-3), respectively. Bootstrap values and BPP are indicated above or below each branch. The position of the strain of *A.* *phagocytophilum* characterized in the present study is highlighted in a gray box

**Fig. 4:**
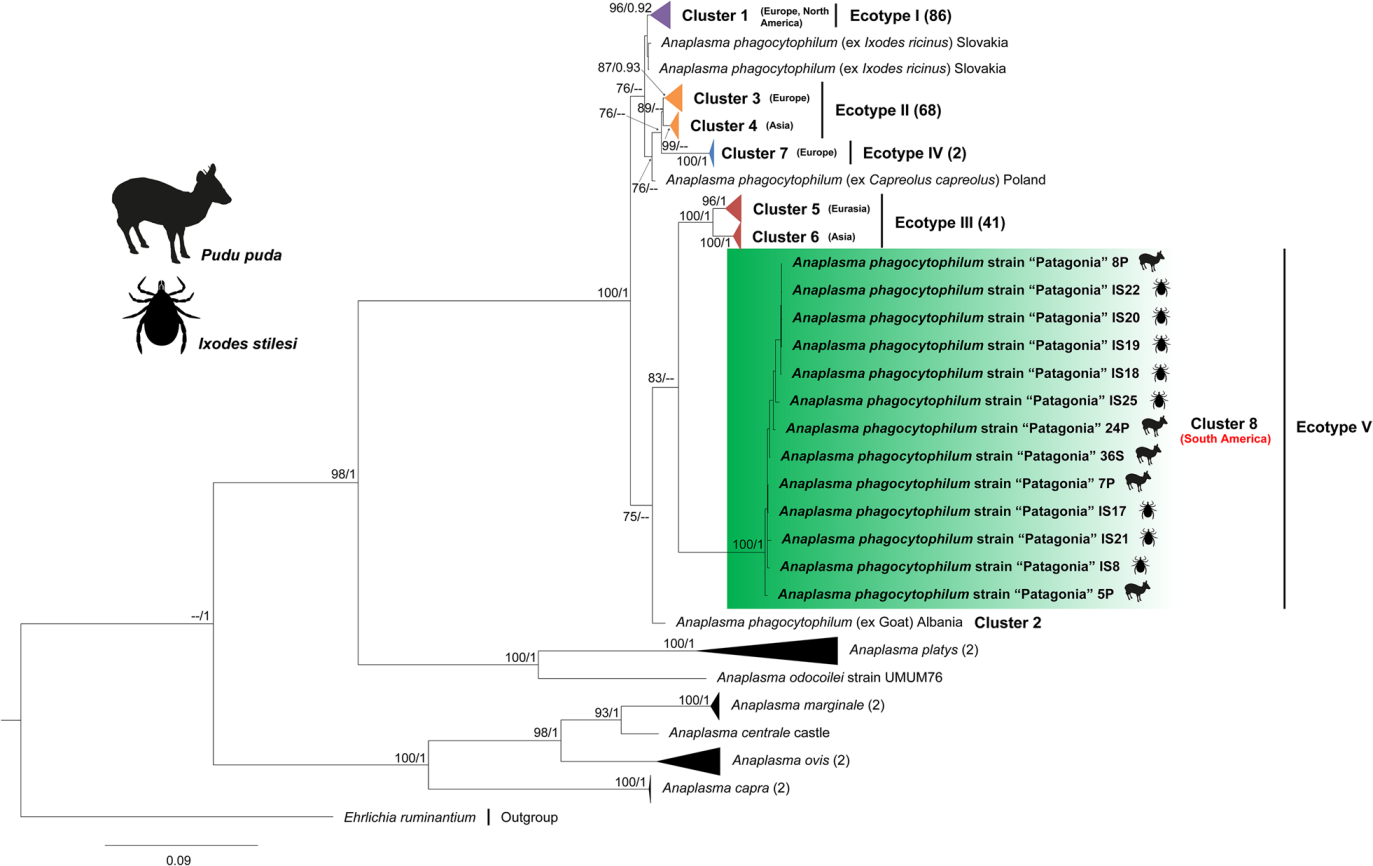
ML and BI consensus tree inferred for a subset of *Anaplasma* spp., using 226 sequences of the *groEL* gene, and an alignment length of 1224 bp. Best-fit evolutionary models calculated for the ML and BI methods were TIM + F + G4 (position-1); TN + F + G4 (position-2); and K3Pu + F + G4 (position-3); and *M*_45_, *M*_136_, *M*_142_, *M*_130_, *M*_139_, *M*_185_ (position-1); *M*_81_, *M*_40_ (position-2); *M*_15_, *M*_50_, *M*_85_, *M*_122_, *M*_90_ (position-3), respectively. Bootstrap values and BPP are indicated above or below each branch. Colors for ecotypes I, II, III and IV were assigned according to Jaarsma et al. [8]. The position of the strain of *A.* *phagocytophilum* characterized in the present study is highlighted in a green box

Phylogenies inferred for the three loci positioned *Anaplasma* genotypes retrieved from *I*. *stilesi* and pudu blood into the *A*. *phagocytophilum* clade, forming a monophyletic group (Figs. 2, 3, 4). In particular, the *groEL* phylogeny placed our genotypes in an independent clade related to ecotype III of *A*. *phagocytophilum* [1] (Fig. 4).

For the *groEL* gene, the average sequence divergence calculated within ecotypes was always less than the average sequence divergence calculated among them, including the ecotype characterized in this study (Table 3). Collectively, the genetic evidence provided by our study points to the finding of a fifth *A.* *phagocytophilum* ecotype, for which the name *A*. *phagocytophilum* strain “Patagonia” is proposed. GenBank accession numbers generated in this study are available in Additional file 1: Tables S1, S2).

**Table 3:** Average sequence divergence within ecotypes and among ecotypes calculated on the basis of corrected pairwise distances for a subset of *A.* *phagocytophilum* *groEl* gene sequences (936 bp)

Ecotype	Ecotype				
	I	II	III	IV	V
I	*0.006007*				
II	0.020384	*0.006410*			
III	0.068461	0.060800	*0.010504*		
IV	0.040475	0.034799	0.065253	*0.000001*	
V	0.090030	0.080672	0.080836	0.079975	*0.003386*

Values highlighted in italics are average sequence divergence within ecotypes

## Discussion

Tick-borne bacteria, including *A*. *phagocytophilum*, are geographically expanding, probably due to climate change and anthropogenic landscape perturbation, both factors that favor the spread of their vectors synergically [13, 60]. Although *A*. *phagocytophilum* was previously thought to be a single bacterial species [61], recent phylogenetic reconstructions have revealed a complex of lineages with different pathogeny, geographical distribution, reservoirs and vectors [1]; nevertheless, host range, zoonotic potential and transmission dynamics of this bacterium are still incompletely solved [1, 7, 8, 11].

Based on average divergence of partial *groEL* sequences (Table 3) and strongly supported phylogenies for *rrs*, *gltA*, and *groEL,* in this study we identified a novel genovariant of *A. phagocytophilum* associated with pudues, for which the name “Patagonia” is proposed (Figs. 2, 3, 4). Accordingly, this genovariant has been designated as the ecotype V (cluster 8) of *A*. *phagocytophilum*, which constitutes the first ecotype of this species complex described for South America. Variants of *A*. *phagocytophilum* are adapted to different hosts and vector species, therefore configuring different enzootic cycles [1, 13]. The fact that *A*. *phagocytophilum* strain “Patagonia” conforms an additional ecotype suggests that the eco-epidemiology of this novel strain differs from those of the northern latitudes.

Cervids such as roe deer (*Capreolus* *capreolus*), red deer (*Cervus* *elaphus*), white-tailed deer (*Odocoileus* *virginianus*), fallow deer (*Dama* *dama*), sika deer (*Cervus* *nippon*) and their associated ticks (*I.* *ricinus* and *I.* *scapularis*) are implicated in the maintenance of endemic cycles of some *A*. *phagocytophilum* variants (e.g. Ap-V1, B, J, S, W) in northern latitudes [13, 62–65]. In contrast, previous knowledge on *A*. *phagocytophilum* in South American deer species is vague, limited only to Brazil, and does not support its classification within any ecotype. For example, in their study on the brown brocket deer (*Mazama* *gouazoubira*), Silveira et al. [15] could not discriminate whether *A*. *phagocytophilum* or *A*. *platys* caused the infection using PCR and sequencing protocols. However, a posterior survey revealed that *A*. *phagocytophilum* would be circulating in brown brocket deer [23]. On the other hand, exposure to *A*. *phagocytophilum* in Brazilian marsh deer (*Blastocerus* *dichotomus*) has been reported using indirect immunofluorescence assays [14]. As far as we know, our study is the first multigenic detection of *A*. *phagocytophilum* DNA in pudu and *I*. *stilesi*.

Records of *A*. *phagocytophilum* in South American mammals include rodents (*Cavia* sp. and *Calomys* *cerqueirai*), peccary (*Tayassu* *pecari* and *Pecari* *tajacu*), sloths (*Bradypus* *tridactylus*) and coati (*Nasua* *nasua*) [17, 66, 67]. However, due to the use of short fragments of the *rrs* and *groEL* genes for identification, it is difficult to state whether the *Anaplasma* DNA detected in these mammals corresponded to *A*. *phagocytophilum* or not. While reports of *A*. *phagocytophilum* on South American cervids are few, other *Anaplasma* spp. have been recorded in deer in Brazil, such as *Anaplasma* *bovis* and *Anaplasma* sp. in red brocket deer (*Mazama* *americana*); *A*. *bovis*, *Anaplasma* *marginale* and *A*. *platys* in marsh deer; and *A*. *marginale* in brown brocket deer [14–17]. Likewise, the records in South America include *A*. *platys*, *Anaplasma* *odocoilei*, *A*. *marginale* and “*Candidatus* Anaplasma boleense” in marsh deer in Argentina [19], and *Anaplasma* sp. Mazama genotype in brown brocket deer in Uruguay [18].

In Chile, evidence of *A*. *phagocytophilum* is incipient. Indeed, infection by this bacterium has been reported in horses [68]. However, these results deserve further investigation, since the use of *A.* *phagocytophilum*-specific primers did not yield positive reactions, and the occurrence of a vector in the area where positive animals were detected is unknown. Further reports of *Anaplasma* spp. in Chile include *A*. *platys* in dogs, Andean foxes (*Lycalopex* *culpaeus*), the South American gray fox (*Lycalopex* *griseus*) [69] and hard ticks (*Rhipicephalus* *sanguineus* sensu lato). An *Anaplasma*-like agent has also been detected in seabird soft ticks (*Ornithodoros* *spheniscus*) [70]. Moreover, serological evidence of exposure to *Anasplasma* sp. has been recorded in dogs [71] and humans [71–74]. Our results thus expand current knowledge on vertebrate hosts of *A*. *phagocytophilum* in the continent.

There is no standardized approach for investigating the genetic diversity and population structure of *Anaplasma* species. Although the *rrs*, *gltA* and *groEL* markers used in this study are currently the most appropriate loci for the genetic characterization of *Anaplasma* spp. [1], *rrs* and *groEL* are conserved and do not have sufficient resolution to segregate some groups when short fragments are analyzed, even in different species of the genus. Therefore, the sequenced fragments must be long enough [1, 6]. Based on the above argument, our phylogenetic analyses did not include sequences shorter than 600 bp.

Previous studies found that the *groEL* gene may delimit lineages (ecotypes, clusters, groups) of *A*. *phagocytophilum* [1, 7, 8, 11]. Moreover, the discrimination capacity among lineages has improved due to the progressive increase in taxon sampling and the size of the sequences employed in the analyses [1]. Recently, a population study recovered ecotypes I, II, III and IV (mentioned by Jahfari et al. [7] and Jaarsma et al. [8]) as monophyletic but without statistical support for ecotypes I and II [1]. It is worth noting that ecotype IV was designated after including only one sequence in those analyses, and its monophyly was not assessed [1]. In addition, cluster 3 (paraphyletic within ecotype II) lacked statistical support (Electronic Supplementary Material Figure S4. in Rar et al. [1]). Thus, methodological factors, such as the inclusion of an outgroup [10, 75], longer alignments, denser taxon sampling [1, 11] and the application of phylogenetic inferences (BI, ML) [39–41], may circumscribe with higher confidence the monophyly and evolutionary relationships of ecotypes and subclades within *A*. *phagocytophilum*, as shown in our study.

Applying the above referred methods, ecotypes I, II and IV were depicted as monophyletic lineages with high statistical support (Fig. 4). In particular, ecotype II was only recovered with high support in ML analyses (92% of bootstrap), yet the cluster 3 (Europe) belonging to this ecotype represents a monophyletic group with confident support (0.94/89) (Fig. 4). Our results differ from those of other studies that described these monophyletic groups based on an eco-epidemiological approach without considering systematics [1, 7, 8, 11]. Undoubtedly, ecotype II and cluster 3 represent natural assemblages, but our study shows them now as also phylogenetically supported. Herein described ecotype V was moderately supported in the *groEL*-based ML inference (81% of ultrafast-bootstrap) and closely related to ecotype III (Fig. 4), which is integrated by variants of *A*. *phagocytophilum* related to small mammals and ticks (Additional file 1: Table S2) [1]. However, the phylogenetic position of the ecotypes should be re-evaluated as new members of the *A. phagocytophilum* complex are discovered.

The presence of *A*. *phagocytophilum* DNA does not conclusively confirm the role of pudues and *I*. *stilesi* in the epidemiology of this bacterium or any clinical impact on pudu health. However, the fact that *P*. *puda* is the sole deer that currently inhabits the areas from which positive animals for this bacterium were recorded [76] strengthens the notion that this cervid could be reservoir of *A*. *phagocytophilum* strain “Patagonia.” In addition, considering the role of *Ixodes* spp. as vectors of *Anaplasma* spp. in the Northern Hemisphere cervids [1], *I*. *stilesi* and *I*. *taglei*, two species that commonly parasitize pudues [22], represent potential vectors of *A*. *phagocytophilum* strain “Patagonia.” However, our hypotheses should be tested in experimental studies. Meanwhile, the epidemiological cycle of *A*. *phagocytophilum* strain “Patagonia” remains unknown.

## Conclusions

We report the presence of and ecotype of *A.* *phagocytophilum* for the first time in South America. The genetic evidence showed conclusively that the *A*. *phagocytophilum* found in this study is a unique variant, and the name *A*. *phagocytophilum* strain “Patagonia” is tentatively proposed. The study of the enzootic cycle of *A*. *phagocytophilum* strain “Patagonia” is now essential to establish its zoonotic potential and health impact on pudues and further species, such as domestic ruminants. Furthermore, because some variants of *A*. *phagocytophilum* are infectious agents of public and veterinary health concern, the detection of this bacterium in Chile deserves further attention. Future research should define a standardized approach for genetically characterizing members of *Anaplasma* genus that would afford reliable comparisons, as recommended in Rar et al. [1]. Finally, these findings bring insight into the genetic diversity and ecology of *A*. *phagocytophilum*.

## Supplementary Information

**Additional file 1: Table S1.** GenBank accession numbers of the sequences used for* Anaplasma phagocytophilum* *rrs* and* gltA* phylogenies. Sequences generated in this study are highlighted in bold.** Table S2.** GenBank accession numbers of the sequences used for* Anaplasma phagocytophilum groEL* phylogeny. Sequences generated in this study are highlighted in bold.

## Data Availability

GenBank accession numbers generated in this study are available in Additional files 1: Tables S1 and S2.

## Abbreviations

BI: Bayesian inference
BLAST: Basic local alignment search tool
*gltA*: Citrate synthase gene
*GAPDH*: Glyceraldehyde-3-phosphate dehydrogenase gene
*groEL*: Heat-shock operon
MAFFT: Multiple alignment using fast Fourier transform
ML: Maximum likelihood
MCMC: Markov chain Monte Carlo
rRNA: Ribosomal ribonucleic acid
*rrs*: 16S rRNA gene

## References

[1] Rar V, Tkachev S, Tikunova N (2021). Genetic diversity of *Anaplasma* bacteria: twenty years later. Infect Genet Evol.

[2] Atif FA (2016). Alpha proteobacteria of genus *Anaplasma* (*Rickettsiales*: *Anaplasmataceae*): epidemiology and characteristics of *Anaplasma* species related to veterinary and public health importance. Parasitology.

[3] Battilani M, de Arcangeli S, Balboni A, Dondi F (2017). Genetic diversity and molecular epidemiology of *Anaplasma*. Infect Genet Evol.

[4] Dumler JS, Barbet AF, Bekker CP, Dasch GA, Palmer GH, Ray SC (2001). Reorganization of genera in the families *Rickettsiaceae* and *Anaplasmataceae* in the order *Rickettsiales*: unification of some species of *Ehrlichia* with *Anaplasma*, *Cowdria* with *Ehrlichia* and *Ehrlichia* with *Neorickettsia*, descriptions of six new species combinations and designation of *Ehrlichia*
*equi* and “HGE agent” as subjective synonyms of *Ehrlichia*
*phagocytophila*. Int J Syst Evol Microbiol.

[5] Matei IA, Estrada-Peña A, Cutler SJ, Vayssier-Taussat M, Varela-Castro L, Potkonjak A (2019). A review on the eco-epidemiology and clinical management of human granulocytic anaplasmosis and its agent in Europe. Parasit Vectors.

[6] Caudill MT, Brayton KA (2022). The use and limitations of the 16S rRNA sequence for species classification of *Anaplasma* samples. Microorganisms.

[7] Jahfari S, Coipan EC, Fonville M, van Leeuwen AD, Hengeveld P, Heylen D (2014). Circulation of four *Anaplasma*
*phagocytophilum* ecotypes in Europe. Parasit Vectors.

[8] Jaarsma RI, Sprong H, Takumi K, Kazimirova M, Silaghi C, Mysterud A (2019). *Anaplasma*
*phagocytophilum* evolves in geographical and biotic niches of vertebrates and ticks. Parasit Vectors.

[9] Palys T, Nakamura LK, Cohan FM (1997). Discovery and classification of ecological diversity in the bacterial world: the role of DNA sequence data. Int J Syst Bacteriol.

[10] Cohan FM (2001). Bacterial species and speciation. Syst Biol.

[11] Rar V, Yakimenko V, Tikunov A, Makenov M, Epikhina T, Tancev A (2020). Genetic variability of *Anaplasmataceae* circulating in small mammals and ticks in an *Ixodes*
*persulcatus*/*Ixodes*
*trianguliceps* sympatric area in Russian Siberia. Ticks Tick Borne Dis.

[12] Remesar S, Prieto A, García-Dios D, López-Lorenzo G, Martínez-Calabuig N, Díaz-Cao JM (2022). Diversity of *Anaplasma* species and importance of mixed infections in roe deer from Spain. Transbound Emerg Dis.

[13] Dugat T, Lagrée A-C, Maillard R, Boulouis H-J, Haddad N (2015). Opening the black box of *Anaplasma*
*phagocytophilum* diversity: current situation and future perspectives. Front Cell Infect Microbiol.

[14] Sacchi ABV, Duarte JMB, André MR, Machado RZ (2012). Prevalence and molecular characterization of *Anaplasmataceae* agents in free-ranging Brazilian marsh deer (*Blastocerus*
*dichotomus*). Comp Immunol Microbiol Infect Dis.

[15] Silveira JAG, Rabelo EML, Ribeiro MFB (2012). Molecular detection of tick-borne pathogens of the family *Anaplasmataceae* in Brazilian brown brocket deer (*Mazama*
*gouazoubira*, Fischer, 1814) and marsh deer (*Blastocerus*
*dichotomus*, Illiger, 1815). Transbound Emerg Dis.

[16] Mongruel ACB, Benevenute JL, André MR, de Carrasco AOT, Machado RZ, Seki MC (2017). Molecular characterization of *Anaplasma* sp. in free-living gray brockets (*Mazama*
*gouazoubira*). Vector Borne Zoonotic Dis..

[17] Soares HS, Marcili A, Barbieri ARM, Minervino AHH, Malheiros AF, Gennari SM (2017). Novel *Anaplasma* and *Ehrlichia* organisms infecting the wildlife of two regions of the Brazilian Amazon. Acta Trop.

[18] Félix ML, Armúa-Fernández MT, Parodi P, Bazzano V, Mangold AJ, Venzal JM (2020). Detection of a putative novel genotype of *Anaplasma* in gray-brocket deer (*Mazama*
*gouazoubira*) from Uruguay. Exp Appl Acarol.

[19] Orozco MM, Argibay HD, Minatel L, Guillemi EC, Berra Y, Schapira A (2020). A participatory surveillance of marsh deer (*Blastocerus*
*dichotomus*) morbidity and mortality in Argentina: first results. BMC Vet Res.

[20] Hidalgo-Hermoso E, Cabello J, Novoa-Lozano I, Celis S, Ortiz C, Kemec I (2022). Molecular detection and characterization of hemoplasmas in the *Pudu* (*Pudu*
*puda*), a native cervid from Chile. J Wildl Dis.

[21] Silva-Rodríguez E, Pastore H, Jiménez J. *Pudu**puda*. In: International Union for Conservation of Nature, editor. The IUCN Red List of threatened species. 2016. 10.2305/IUCN.UK.2016-1.RLTS.T18848A22164089.en.

[22] Nava S, Venzal JM, González-Acuña D, Martins TF, Guglielmone AA (2017). Ticks of the Southern Cone of America.

[23] Silveira JAG, Rabelo EML, Lima PCS, Chaves BN, Ribeiro MFB (2014). Post-mortem hemoparasite detection in free-living Brazilian brown brocket deer (*Mazama*
*gouazoubira*, Fischer 1814). Rev Bras Parasitol Vet.

[24] Khare P, Raj V, Chandra S, Agarwal S (2014). Quantitative and qualitative assessment of DNA extracted from saliva for its use in forensic identification. J Forensic Dent Sci.

[25] Birkenheuer AJ, Levy MG, Breitschwerdt EB (2003). Development and evaluation of a seminested PCR for detection and differentiation of *Babesia*
*gibsoni* (Asian genotype) and *B.*
*canis* DNA in canine blood samples. J Clin Microbiol.

[26] Mangold AJ, Bargues MD, Mas-Coma S (1998). Mitochondrial 16S rDNA sequences and phylogenetic relationships of species of *Rhipicephalus* and other tick genera among Metastriata (Acari: Ixodidae). Parasitol Res.

[27] Anderson BE, Dawson JE, Jones DC, Wilson KH (1991). *Ehrlichia*
*chaffeensis*, a new species associated with human ehrlichiosis. J Clin Microbiol.

[28] Paddock CD, Sumner JW, Shore GM, Bartley DC, Elie RC, McQuade JG (1997). Isolation and characterization of *Ehrlichia*
*chaffeensis* strains from patients with fatal ehrlichiosis. J Clin Microbiol.

[29] Kawahara M, Rikihisa Y, Isogai E, Takahashi M, Misumi H, Suto C (2004). Ultrastructure and phylogenetic analysis of “*Candidatus* Neoehrlichia mikurensis” in the family *Anaplasmataceae*, isolated from wild rats and found in *Ixodes*
*ovatus* ticks. Int J Syst Evol Microbiol.

[30] Liz JS, Anderes L, Sumner JW, Massung RF, Gern L, Rutti B (2000). PCR detection of granulocytic ehrlichiae in *Ixodes*
*ricinus* ticks and wild small mammals in western Switzerland. J Clin Microbiol.

[31] Huang H, Unver A, Perez MJ, Orellana NG, Rikihisa Y (2005). Prevalence and molecular analysis of *Anaplasma*
*platys* in dogs in Lara, Venezuela. Braz J Microbiol.

[32] Tabara K, Arai S, Kawabuchi T, Itagaki A, Ishihara C, Satoh H (2007). Molecular survey of *Babesia*
*microti*, *Ehrlichia* species and *Candidatus* Neoehrlichia mikurensis in wild rodents from Shimane Prefecture, Japan. Microbiol Immunol.

[33] Gofton AW, Doggett S, Ratchford A, Ryan U, Irwin P (2016). Phylogenetic characterisation of two novel *Anaplasmataceae* from Australian *Ixodes*
*holocyclus* ticks: “*Candidatus* Neoehrlichia australis” and “*Candidatus* Neoehrlichia arcana”. Int J Syst Evol Microbiol.

[34] Inokuma H, Brouqui P, Drancourt M, Raoult D (2001). Citrate synthase gene sequence: a new tool for phylogenetic analysis and identification of *Ehrlichia*. J Clin Microbiol.

[35] Ewing B, Green P (1998). Base-calling of automated sequencer traces using Phred. II. Error probabilities. Genome Res.

[36] Ewing B, Hillier LD, Wendl MC, Green P (1998). Base-calling of automated sequencer traces using Phred. I. Accuracy assessment. Genome Res.

[37] Katoh K, Standley DM (2013). MAFFT multiple sequence alignment software version 7: improvements in performance and usability. Mol Biol Evol.

[38] Criscuolo A, Gribaldo S (2010). BMGE (Block Mapping and Gathering with Entropy): a new software for selection of phylogenetic informative regions from multiple sequence alignments. BMC Evol Biol.

[39] Yang Z, Rannala B (1997). Bayesian phylogenetic inference using DNA sequences: a Markov chain Monte Carlo method. Mol Biol Evol.

[40] Rannala B, Yang Z (1996). Probability distribution of molecular evolutionary trees: a new method of phylogenetic inference. J Mol Evol.

[41] Felsenstein J (1981). Evolutionary trees from DNA sequences: a maximum likelihood approach. J Mol Evol.

[42] Ronquist F, Teslenko M, van der Mark P, Ayres DL, Darling A, Höhna S (2012). MrBayes 3.2: efficient Bayesian phylogenetic inference and model choice across a large model space. Syst Biol.

[43] Nguyen L-T, Schmidt HA, von Haeseler A, Minh BQ (2015). IQ-TREE: a fast and effective stochastic algorithm for estimating maximum-likelihood phylogenies. Mol Biol Evol.

[44] Yang Z (1996). Maximum-likelihood models for combined analyses of multiple sequence data. J Mol Evol.

[45] Kainer D, Lanfear R (2015). The effects of partitioning on phylogenetic inference. Mol Biol Evol.

[46] Lanfear R, Calcott B, Ho SYW, Guindon S (2012). PartitionFinder: combined selection of partitioning schemes and substitution models for phylogenetic analyses. Mol Biol Evol.

[47] Kalyaanamoorthy S, Minh BQ, Wong TKF, von Haeseler A, Jermiin LS (2017). ModelFinder: fast model selection for accurate phylogenetic estimates. Nat Methods.

[48] Minh BQ, Nguyen MAT, von Haeseler A (2013). Ultrafast approximation for phylogenetic Bootstrap. Mol Biol Evol.

[49] Huelsenbeck JP (2004). Bayesian phylogenetic model selection using reversible jump Markov chain Monte Carlo. Mol Biol Evol.

[50] Rambaut A, Drummond AJ, Xie D, Baele G, Suchard MA (2018). Posterior summarization in Bayesian phylogenetics using Tracer 1.7. Syst Biol.

[51] Huelsenbeck JP, Rannala B (2004). Frequentist properties of Bayesian posterior probabilities of phylogenetic trees under simple and complex substitution models. Syst Biol.

[52] Schwarz G (1978). Estimating the dimension of a model. Ann Stat.

[53] Silvestro D, Michalak I (2012). raxmlGUI: a graphical front-end for RAxML. Org Divers Evol.

[54] Edler D, Klein J, Antonelli A, Silvestro D (2021). raxmlGUI 2.0: a graphical interface and toolkit for phylogenetic analyses using RAxML. Methods Ecol Evol.

[55] Stamatakis A (2014). RAxML version 8: a tool for phylogenetic analysis and post-analysis of large phylogenies. Bioinformatics.

[56] Guglielmone AA, Venzal JM, González-Acuña D, Nava S, Hinojosa A, Mangold AJ (2006). The phylogenetic position of *Ixodes*
*stilesi* Neumann, 1911 (Acari: Ixodidae): morphological and preliminary molecular evidences from 16S rDNA sequences. Syst Parasitol.

[57] Kolo AO, Collins NE, Brayton KA, Chaisi M, Blumberg L, Frean J (2020). *Anaplasma*
*phagocytophilum* and other *Anaplasma* spp. in various hosts in the Mnisi community, Mpumalanga province, South Africa. Microorganisms..

[58] Alberdi P, Ayllón N, Cabezas-Cruz A, Bell-Sakyi L, Zweygarth E, Stuen S (2015). Infection of *Ixodes* spp. tick cells with different *Anaplasma*
*phagocytophilum* isolates induces the inhibition of apoptotic cell death. Ticks Tick Borne Dis..

[59] Fujii Y, Shoji Y, Kanda T, Kishida A, Asano M, Kishida K (2019). The first canine *Anaplasma*
*phagocytophilum* infection in western Japan: a case report. J Anim Clin Med.

[60] Rikihisa Y (2011). Mechanisms of obligatory intracellular infection with *Anaplasma*
*phagocytophilum*. Clin Microbiol Rev.

[61] Stuen S, Granquist EG, Silaghi C (2013). *Anaplasma*
*phagocytophilum*—a widespread multi-host pathogen with highly adaptive strategies. Front Cell Infect Microbiol.

[62] Massung RF, Mather TN, Levin ML (2006). Reservoir competency of goats for the Ap-Variant 1 strain of *Anaplasma*
*phagocytophilum*. Infect Immun.

[63] Silaghi C, Hamel D, Thiel C, Pfister K, Passos LMF, Rehbein S (2011). Genetic variants of *Anaplasma*
*phagocytophilum* in wild caprine and cervid ungulates from the Alps in Tyrol, Austria. Vector Borne Zoonotic Dis.

[64] Overzier E, Pfister K, Thiel C, Herb I, Mahling M, Silaghi C (2013). *Anaplasma*
*phagocytophilum* in questing *Ixodes*
*ricinus* ticks: comparison of prevalences and partial 16S rRNA gene variants in urban, pasture, and natural habitats. Appl Environ Microbiol.

[65] Overzier E, Pfister K, Herb I, Mahling M, Böck G, Silaghi C (2013). Detection of tick-borne pathogens in roe deer (*Capreolus*
*capreolus*), in questing ticks (*Ixodes*
*ricinus*), and in ticks infesting roe deer in southern Germany. Ticks Tick Borne Dis.

[66] Benevenute JL, Dumler JS, Ogrzewalska M, Roque ALR, Mello VVC, de Sousa KCM (2017). Assessment of a quantitative 5′ nuclease real-time polymerase chain reaction using *groEL* gene for *Ehrlichia* and *Anaplasma* species in rodents in Brazil. Ticks Tick Borne Dis.

[67] de Sousa KCM, Calchi AC, Herrera HM, Dumler JS, Barros-Battesti DM, Machado RZ (2017). *Anaplasmataceae* agents among wild mammals and ectoparasites in Brazil. Epidemiol Infect.

[68] Hurtado C, Torres R, Pérez-Macchi S, Sagredo K, Uberti B, de Souza Zanatto DC (2020). Serological and molecular detection of *Anaplasma*
*phagocytophilum* in thoroughbred horses from Chilean racecourses. Ticks Tick Borne Dis.

[69] di Cataldo S, Cevidanes A, Ulloa-Contreras C, Hidalgo-Hermoso E, Gargano V, Sacristán I (2021). Mapping the distribution and risk factors of *Anaplasmataceae* in wild and domestic canines in Chile and their association with *Rhipicephalus*
*sanguineus* species complex lineages. Ticks Tick Borne Dis.

[70] Muñoz-Leal S, Lopes MG, Marcili A, Martins TF, González-Acuña D, Labruna MB (2019). *Anaplasmataceae*, *Borrelia* and *Hepatozoon* agents in ticks (Acari: Argasidae, Ixodidae) from Chile. Acta Trop.

[71] Acosta-Jamett G, Weitzel T, López J, Alvarado D, Abarca K (2020). Prevalence and risk factors of antibodies to *Anaplasma* spp. in Chile: a household-based cross-sectional study in healthy adults and domestic dogs. Vector Borne Zoonotic Dis..

[72] Abarca K, López J, Perret C, Guerrero J, Godoy P, Veloz A (2007). *Anaplasma*
*platys* in dogs, Chile. Emerg Infect Dis.

[73] Weinborn AR, Zanelli GM, López SÓ, Pau VN, Valdés PF (2018). Anticuerpos anti-*Anaplasma* spp en población de riesgo ocupacional de un hospital veterinario. Rev Investig Vet Peru.

[74] Conejeros Ortiz C, Rodríguez Jorquera P. Diagnóstico serológico de *Anaplasma**phagocytophilum* en caballos fina sangre de carrera pertenecientes al Valparaíso Sporting Club Viña del Mar. Saarbrücken: Editorial Académica Española; 2013.

[75] Hennig W (1965). Phylogenetic systematics. Annu Rev Entomol.

[76] Iriarte A. Mamíferos de Chile. 1st edn. Santiago: Lynx Edicions; 2008.

[77] Muñoz-Leal S, Silva-De-La-Fuente MC, Barros-Battesti DM, Guglielmone AA, Venzal JM, Nava S (2021). In memoriam: a eulogy for Daniel González-Acuña, 1963–2020. Rev Bras Parasitol Vet.

