# Supplementary material for: Wild deer (Pudu puda) from Chile harbor a novel ecotype of Anaplasma phagocytophilum
Source: Parasit Vectors. 2023 Jan 27;16:38. doi: 10.1186/s13071-023-05657-9 (PMC9883915; doi:10.1186/s13071-023-05657-9)
Supplement: Supplementary file 1 — Additional file 1: Table S1. GenBank accession numbers of the sequences used for Anaplasma phagocytophilum rrs and gltA phylogenies. Sequences generated in this study are highlighted in bold. Table S2. GenBank accession numbers of the sequences used for Anaplasma phagocytophilum groEL phylogeny. Sequences generated in this study are highlighted in bold. [file 13071_2023_5657_MOESM1_ESM.docx]

**Additional file 1: Table S1.** GenBank accession numbers of the sequences used for *Anaplasma phagocytophilum* 16S rRNA and *gltA* phylogenies. Sequences generated in this study are highlighted in bold.

| **Sequence name** | **GenBank  accession number** | **Host** | **Country** |
| --- | --- | --- | --- |
| **16S rRNA** | | | |
| *Anaplasma phagocytophilum* strain HZ2 | CP006616 | Human | USA |
| *Anaplasma phagocytophilum* strain Webster | NR044762 | Human | USA |
| *Anaplasma phagocytophilum* strain Dog2 | CP006618 | Dog | USA |
| *Anaplasma phagocytophilum* strain Susy | AY527213 | Horse | Sweden |
| *Anaplasma phagocytophilum* strain ApMuc02c | JX173652 | *Ixodes ricinus* | Australia |
| *Anaplasma phagocytophilum* isolate D2_2 genotype Aph1 | MK814406 | Dog | South Africa |
| *Anaplasma phagocytophilum* strain ApMuc01c | JX173651 | Dog | Germany |
| *Anaplasma phagocytophilum* isolate F9_16S | MW677507 | *Ixodes tapirus* | Panama |
| *Anaplasma phagocytophilum* isolate F10_16S | MW677508 | *Ixodes tapirus* | Panama |
| *Anaplasma phagocytophilum* isolate CAHU-HGE2 | AF093789 | Human | USA |
| *Anaplasma phagocytophilum* isolate MR-23 | KP276588 | *Ixodes pacificus* | USA |
| *Anaplasma phagocytophilum* isolate CASTIL | AF172166 | Horse | USA |
| *Anaplasma phagocytophilum* strain ES34 | AB196720 | *Cervus nippon yesoensis* | Japan |
| *Anaplasma* sp. Ac52D | AB588974 | Deer | Japan |
| *Anaplasma phagocytophilum* strain AAIK4 | KR611719 | *Apodemus agrarius* | South Korea |
| *Anaplasma phagocytophilum* isolate Nov-Ip355 | HM366580 | *Ixodes persulcatus* | Russia |
| ***Anaplasma phagocytophilum* 36S** | **OP579238** | ***Pudu puda*** | **Chile** |
| ***Anaplasma phagocytophilum* 45S** | **OP579239** | ***Pudu puda*** | **Chile** |
| ***Anaplasma phagocytophilum* 5P** | **OP579240** | ***Pudu puda*** | **Chile** |
| ***Anaplasma phagocytophilum* 8P** | **OP579242** | ***Pudu puda*** | **Chile** |
| ***Anaplasma phagocytophilum* 7P** | **OP579241** | ***Pudu puda*** | **Chile** |
| ***Anaplasma phagocytophilum* IS25** | **OP579248** | ***Ixodes stilesi*** | **Chile** |
| ***Anaplasma phagocytophilum* IS19** | **OP579246** | ***Ixodes stilesi*** | **Chile** |
| ***Anaplasma phagocytophilum* IS22** | **OP579247** | ***Ixodes stilesi*** | **Chile** |
| ***Anaplasma phagocytophilum* 24P** | **OP579243** | ***Pudu puda*** | **Chile** |
| ***Anaplasma phagocytophilum* IS18** | **OP579245** | ***Ixodes stilesi*** | **Chile** |
| ***Anaplasma phagocytophilum* IS17** | **OP579244** | ***Ixodes stilesi*** | **Chile** |
| *Anaplasma platys* | LC269820 | Dog | Zambia |
| *Anaplasma platys* | AY530806 | Dog | Spain |
| *Anaplasma odocoilei* strain UMUM76 | JX876644 | *Odocoileus virginianus* | USA |
| *Anaplasma bovis* | AB196475 | *Haemaphysalis longicornis* | Japan |
| *Anaplasma bovis* | U03775 | Bovine | Senegal |
| *Anaplasma* sp. clone ES1 | KC811530 | *Elephantulus myurus* | South Africa |
| *Anaplasma* sp. clone genotype Mazama | MN817942 | *Mazama gouazoubira* | Uruguay |
| *Anaplasma ovis* isolate OVI | AF414870 | Goat | South Africa |
| *Anaplasma ovis* | AJ633049 | Goat | China |
| *Anaplasma centrale* strain vaccine | AF414868 |  | South Africa |
| *Anaplasma marginale* isolate Lushi | AJ633048 | Cattle | China |
| *Anaplasma marginale* | AF311303 | Bovine | USA |
| *Anaplasma marginale* from Uruguay | AF414877 |  | Uruguay |
| *Ehrlichia ruminantium* strain Welgevonden | NR074155 |  | South Africa |
| ***gltA*** | | | |
| ***Anaplasma phagocytophilum* IS17** | **OP585594** | ***Ixodes stilesi*** | **Chile** |
| ***Anaplasma phagocytophilum* IS25** | **OP585602** | ***Ixodes stilesi*** | **Chile** |
| ***Anaplasma phagocytophilum* IS19** | **OP585593** | ***Ixodes stilesi*** | **Chile** |
| ***Anaplasma phagocytophilum* 36S** | **OP585597** | ***Pudu puda*** | **Chile** |
| ***Anaplasma phagocytophilum* 7P** | **OP585599** | ***Pudu puda*** | **Chile** |
| ***Anaplasma phagocytophilum* IS20** | **OP585600** | ***Ixodes stilesi*** | **Chile** |
| ***Anaplasma phagocytophilum* IS18** | **OP585598** | ***Ixodes stilesi*** | **Chile** |
| ***Anaplasma phagocytophilum* IS22** | **OP585601** | ***Ixodes stilesi*** | **Chile** |
| ***Anaplasma phagocytophilum* 8P** | **OP585596** | ***Pudu puda*** | **Chile** |
| ***Anaplasma phagocytophilum* 24P** | **OP585595** | ***Pudu puda*** | **Chile** |
| ***Anaplasma phagocytophilum* IS21** | **OP585592** | ***Ixodes stilesi*** | **Chile** |
| ***Anaplasma phagocytophilum* 5P** | **OP585591** | ***Pudu puda*** | **Chile** |
| *Anaplasma phagocytophilum* isolate 96HE158(NY8) | AY464138 |  | USA |
| *Anaplasma phagocytophilum* Webster | AF304136 |  | USA |
| *Anaplasma phagocytophilum* isolate 96HE54 | AY464136 |  | Japan |
| *Anaplasma phagocytophilum* isolate 97E13 | AY464134 |  | USA |
| *Anaplasma phagocytophilum* isolate 97HE97 | AY464137 |  | USA |
| *Anaplasma phagocytophilum* strain HGE1 HGE1_contig2 | APHH01000002 | Human | USA |
| *Anaplasma phagocytophilum* 1602 | AF304138 | Sheep | Spain |
| *Anaplasma phagocytophilum* strain Norway variant2 | CP015376 | Sheep | Norway |
| *Anaplasma phagocytophilum* | AY339602 | *Ixodes persulcatus* | Russian |
| *Anaplasma* sp. clone 1 | JN055361 | *Cervus nippon yasoensis* | Japan |
| *Anaplasma* sp. clone 2 | JN055362 | *Cervus nippon yasoensis* | Japan |
| *Anaplasma* sp. BL099-6 | KJ410280 | *Hyalomma asiaticum* | China |
| *Anaplasma* sp. clone Xinjiang099-11 | JX402608 | *Hyalomma asiaticum* | China |
| *Anaplasma platys* | AY077620 | Dog | Japan |
| *Anaplasma platys* | KR011928 | *Rhipicephalus* sp. | China |
| *Anaplasma platys* | EU516387 | Dog | Brazil |
| "*Candidatus* Anaplasma cinensis" AK-Rm-403 | MH716422 | *Rhipicephalus microplus* | China |
| "*Candidatus* Anaplasma cinensis" AK-Rm-228 | MH716426 | *Rhipicephalus microplus* | China |
| *Anaplasma odocoilei* | DQ020101 | *Odocoileus virginianus* | USA |
| *Anaplasma bovis* isolate Wangmang-goat-62 | MH255920 | Goat | China |
| *Anaplasma* sp. clone 499 | JN588561 | *Procyon lotor* | Japan |
| *Anaplasma marginale* | KX987367 | *Rhipicephalus microplus* | China |
| *Anaplasma marginale* | AF304140 |  | USA |
| *Anaplasma ovis* strain Haibei | CP015994 |  | China |
| *Anaplasma centrale* strain Israel | CP001759 |  | Israel |
| *Anaplasma capra* Zhengxiaocun-50 | MG869310 | Goat | China |
| *Anaplasma capra* AK-Rm-429 | MH716413 | *Rhipicephalus microplus* | China |
| *Ehrlichia canis* | AY647155 |  | Italy |

**Additional file 2: Table S2.** GenBank accession numbers of the sequences used for *Anaplasma phagocytophilum groEL* phylogeny. Sequences generated in this study are highlighted in bold.

| **Sequence name** | **GenBank accession numbers** | **Host** | **Country** | **Cluster** | **Ecotype** |
| --- | --- | --- | --- | --- | --- |
| *Anaplasma phagocytophilum* isolate Omsk-6_Mglar | MN701631 | *Myodes glareolus* | Rusia | 5 | III |
| *Anaplasma phagocytophilum* isolate Omsk-104_Mrut | MN701630 | *Myodes rutilus* | Rusia | 5 | III |
| *Anaplasma phagocytophilum* isolate Omsk-65_Itr_m | MN701644 | *Ixodes trianguliceps* | Rusia | 5 | III |
| *Anaplasma phagocytophilum* isolate Omsk-Tr7 | KF745746 | *Ixodes trianguliceps* | Rusia | 5 | III |
| *Anaplasma phagocytophilum* isolate Omsk-Tr3 | KF745745 | *Ixodes trianguliceps* | Rusia | 5 | III |
| *Anaplasma phagocytophilum* clone Omsk-vole54 | KC583431 | *Myodes rufocanus* | Rusia | 5 | III |
| *Anaplasma phagocytophilum* isolate Omsk-41_Mrut | MN701636 | *Myodes rutilus* | Rusia | 5 | III |
| *Anaplasma phagocytophilum* isolate Omsk-56_Mruf | MN701635 | *Myodes rufocanus* | Rusia | 5 | III |
| *Anaplasma phagocytophilum* isolate Omsk-28_Apagr | MN701632 | *Apodemus agrarius* | Rusia | 5 | III |
| *Anaplasma phagocytophilum* isolate Omsk-Tr17 | KF745747 | *Ixodes trianguliceps* | Rusia | 5 | III |
| *Anaplasma phagocytophilum* clone Omsk-vole121 | KC583432 | *Myodes rutilus* | Rusia | 5 | III |
| *Anaplasma phagocytophilum* isolate 224691FITAFmouse | KF383236 | *Ixodes trianguliceps* | Slovakia | 5 | III |
| *Anaplasma phagocytophilum* isolate 228141LITMGvole | KF383232 | *Ixodes trianguliceps* | Slovakia | 5 | III |
| *Anaplasma phagocytophilum* isolate 227781LITMGvole | KF383235 | *Ixodes trianguliceps* | Slovakia | 5 | III |
| *Anaplasma phagocytophilum* isolate Omsk-51_Mruf | MN701629 | *Myodes rufocanus* | Rusia | 5 | III |
| *Anaplasma phagocytophilum* isolate Omsk-23_Mrut | MN701628 | *Myodes rutilus* | Rusia | 5 | III |
| *Anaplasma phagocytophilum* isolate Omsk-167_Micagr | MN701633 | *Microtus agrestis* | Rusia | 5 | III |
| *Anaplasma phagocytophilum* isolate 220166sMGvole | KF383231 | *Myodes glareolus* | Slovakia | 5 | III |
| *Anaplasma phagocytophilum* isolate Sv-shrew70 | HQ630617 | *Sorex araneus* | Rusia | 5 | III |
| *Anaplasma phagocytophilum* isolate Sv-vole8 | HQ630616 | *Myodes rutilus* | Rusia | 5 | III |
| *Anaplasma phagocytophilum* strain ItalyHU148 | KF031390 | *Myodes glareolus* | Italy | 5 | III |
| *Anaplasma phagocytophilum* isolate 227841LITMGvole | KF383233 | *Ixodes trianguliceps* | Slovakia | 5 | III |
| *Anaplasma phagocytophilum* isolate Omsk-17_Mrut | MN701639 | *Myodes rutilus* | Rusia | 6 | III |
| *Anaplasma phagocytophilum* isolate Omsk-9-13_Mruf | MN609907 | *Myodes rufocanus* | Rusia | 6 | III |
| *Anaplasma phagocytophilum* isolate RUS/Alt14-1625-Ipv | KX980041 | *Ixodes pavlovskyi* | Rusia | 6 | III |
| *Anaplasma phagocytophilum* isolate Nov-Ip456 | HM366570 | *Ixodes persulcatus* | Rusia | 6 | III |
| *Anaplasma phagocytophilum* isolate Irk-Ip625 | HM366571 | *Ixodes persulcatus* | Rusia | 6 | III |
| *Anaplasma phagocytophilum* isolate Nov-vole144 | HQ630614 | *Myodes rufocanus* | Rusia | 6 | III |
| *Anaplasma phagocytophilum* isolate Kh-vole305 | HQ630615 | *Myodes rufocanus* | Rusia | 6 | III |
| *Anaplasma phagocytophilum* isolate Tomsk-Ipr1 | KF701460 | *Ixodes trianguliceps* | Rusia | 6 | III |
| *Anaplasma phagocytophilum* clone Tuva-Ip2947 | KC753764 | *Ixodes persulcatus* | Rusia | 6 | III |
| *Anaplasma phagocytophilum* isolate Tomsk Dr-1 | KY379956 | *Dermacentor reticulatus* | Rusia | 6 | III |
| *Anaplasma phagocytophilum* isolate Tomsk-Ipr2 | KF701461 | *Ixodes persulcatus* | Rusia | 6 | III |
| *Anaplasma phagocytophilum* isolate Tomsk-Ipr3 | KF701462 | *Ixodes persulcatus* | Rusia | 6 | III |
| *Anaplasma phagocytophilum* isolate Tomsk-Ipr4 | KY684729 | *Ixodes persulcatus* | Rusia | 6 | III |
| *Anaplasma phagocytophilum* isolate Tomsk-Ipr6 | KY684731 | *Ixodes persulcatus* | Rusia | 6 | III |
| *Anaplasma phagocytophilum* isolate Tomsk-Ipr8 | KY684733 | *Ixodes persulcatus* | Rusia | 6 | III |
| *Anaplasma phagocytophilum* isolate Tomsk-Ipr5 | KY684730 | *Ixodes persulcatus* | Rusia | 6 | III |
| *Anaplasma phagocytophilum* isolate Tomsk-Ipr7 | KY684732 | *Ixodes persulcatus* | Rusia | 6 | III |
| *Anaplasma phagocytophilum* isolate Omsk-43_Mglar | MN701638 | *Myodes glareolus* | Rusia | 6 | III |
| *Anaplasma phagocytophilum* isolate RUS/Alt14-2442-Ipr/Ipv | MG182152 | *Ixodes persulcatus* | Rusia | 6 | III |
| *Anaplasma phagocytophilum* isolate G22 | AY281818 | *Ixodes ricinus* | Germany | 1 | I |
| *Anaplasma phagocytophilum* isolate 472 | AF478561 | *Capreolus capreolus* | Slovenia | 1 | I |
| *Ehrlichia phagocytophila* #99 | AF383227 | *Capreolus capreolus* | Switzerland | 1 | I |
| *Anaplasma phagocytophilum* isolate 70 | JN005748 | *Capreolus capreolus* | Poland | 1 | I |
| *Anaplasma phagocytophilum* isolate 832 | AF478553 | *Cervus elaphus* | Slovenia | 1 | I |
| *Ehrlichia phagocytophila* | U96730 | Sheep | Great Britain | 1 | I |
| *Ehrlichia phagocytophila* | U96729 | Goat | Great Britain | 1 | I |
| *Anaplasma phagocytophilum* strain c-D3160 | KM215266 | *Rupicapra rupicapra* | Slovenia | 1 | I |
| *Anaplasma phagocytophilum* strain tick-EU431 | KM215251 | *Ixodes ricinus* | Slovenia | 1 | I |
| *Anaplasma phagocytophilum* isolate 2 | AF548386 | Sheep | Norway | 1 | I |
| *Anaplasma phagocytophilum* strain c-D3155 | KM215265 | *Rupicapra rupicapra* | Slovenia | 1 | I |
| *Anaplasma phagocytophilum* isolate GC45 | HM057228 | *Ixodes ricinus* | Spain | 1 | I |
| *Anaplasma phagocytophilum* isolate 0511 | HM057225 | *Cervus elaphus* | Spain | 1 | I |
| *Anaplasma phagocytophilum* strain c-2829 | KM215264 | *Rupicapra rupicapra* | Slovenia | 1 | I |
| *Anaplasma phagocytophilum* isolate 09/78 | HM057232 | *Ixodes ricinus* | Rusia | 1 | I |
| *Anaplasma phagocytophilum* strain bear-9304 | KJ622308 | *Ursus arctos* | Slovenia | 1 | I |
| *Anaplasma phagocytophilum* isolate 474 | AF478563 | *Cervus elaphus* | Slovenia | 1 | I |
| *Anaplasma phagocytophilum* strain bear-9503 | KJ622307 | *Ursus arctos* | Slovenia | 1 | I |
| *Anaplasma phagocytophilum* isolate dog-7425 | EU381151 | Dog | Slovenia | 1 | I |
| *Anaplasma phagocytophilum* isolate G55 | AY281823 | *Ixodes ricinus* | Germany | 1 | I |
| *Anaplasma phagocytophilum* GroESL | EF392724 | *Ixodes ricinus* | Croatia | 1 | I |
| *Anaplasma phagocytophilum* isolate tick-40 | EU381152 | *Ixodes ricinus* | Slovenia | 1 | I |
| *Anaplasma phagocytophilum* strain tick-EU343 | KM215246 | *Ixodes ricinus* | Slovenia | 1 | I |
| *Anaplasma phagocytophilum* isolate 921 | AF478558 | *Capreolus capreolus* | Slovenia | 1 | I |
| *Anaplasma phagocytophilum* isolate 61g | HM057230 | *Ixodes ricinus* | Spain | 1 | I |
| *Anaplasma phagocytophilum* isolate 1 | AF548385 | Sheep | Norway | 1 | I |
| *Anaplasma phagocytophilum* isolate 09/71 | HM057231 | *Ixodes ricinus* | Spain | 1 | I |
| *Anaplasma phagocytophilum* strain c-708 | KM215263 | *Rupicapra rupicapra* | Slovenia | 1 | I |
| *Anaplasma phagocytophilum* isolate W271 | AY281844 | *Ixodes ricinus* | Germany | 1 | I |
| *Anaplasma phagocytophilum* strain tick-EU108 | KM215252 | *Ixodes ricinus* | Slovenia | 1 | I |
| *Anaplasma phagocytophilum* isolate I94 | AY281828 | *Ixodes ricinus* | Germany | 1 | I |
| *Ehrlichia phagocytophila* | AF202895 | *Ixodes ricinus* | Switzerland | 1 | I |
| *Anaplasma phagocytophilum* isolate 3C/2310/1 | HM057224 | *Cervus elaphus* | Spain | 1 | I |
| *Anaplasma phagocytophilum* isolate L6-9 | HM057233 | *Ixodes ricinus* | Russia | 1 | I |
| *Anaplasma phagocytophilum* isolate tick-43 | EU246959 | *Ixodes ricinu* | Slovenia | 1 | I |
| *Anaplasma phagocytophilum* isolate GC19 | HM057227 | *Ixodes ricinus* | Spain | 1 | I |
| *Anaplasma phagocytophilum* strain red-D3217 | KM215262 | *Cervus elaphus* | Slovenia | 1 | I |
| *Anaplasma phagocytophilum* isolate 473 | AF478562 | *Cervus elaphus* | Slovenia | 1 | I |
| *Anaplasma phagocytophilum* isolate 707 | AF478557 | *Cervus elaphus* | Slovenia | 1 | I |
| *Anaplasma phagocytophilum* strain red-D3009 | KM215261 | *Cervus elaphus* | Slovenia | 1 | I |
| *Anaplasma phagocytophilum* isolate 812 groESL | AF478552 | *Cervus elaphus* | Slovenia | 1 | I |
| *Anaplasma phagocytophilum* isolate dog-7414 | EU381150 | Dog | Slovenia | 1 | I |
| *Anaplasma phagocytophilum* | EU184703 | *Sus scrofa* | Slovenia | 1 | I |
| *Anaplasma phagocytophilum* isolate N6 | AY281849 | *Ixodes ricinus* | Germany | 1 | I |
| *Ehrlichia* sp. 'HGE agent' | AF033101 | Human | Slovenia | 1 | I |
| *Anaplasma phagocytophilum* | AF482760 | Horse | Germany | 1 | I |
| *Anaplasma phagocytophilum* strain Strong | AY529490 | Horse | Sweden | 1 | I |
| *Anaplasma phagocytophilum* isolate 163HFIRQ | KF383241 | *Ixodes ricinus* | Slovakia | 1 | I |
| *Anaplasma phagocytophilum* isolate 39FCIRQ | KF383239 | *Ixodes ricinus* | Slovakia | 1 | I |
| *Anaplasma phagocytophilum* isolate 187 | EU860089 | Sheep | France | 1 | I |
| *Anaplasma phagocytophilum* | KF836094 | Dog | Brazil | 1 | I |
| *Anaplasma phagocytophilum* strain Susy | AY529489 | Horse | Sweden | 1 | I |
| *Anaplasma phagocytophilum* strain Fordyce | EF647585 | Horse | USA | 1 | I |
| *Anaplasma phagocytophilum* | DQ680012 | Cat | USA | 1 | I |
| *Anaplasma phagocytophilum* | AY219849 | Dog | USA | 1 | I |
| *Ehrlichia equi* | AF173989 | *Ixodes pacificus* | USA | 1 | I |
| *Ehrlichia equi* | AF173988 | *Neotoma fuscipes* | USA | 1 | I |
| *Anaplasma phagocytophilum* clone KC15 | AY626252 | *Sigmodon hispidus* | USA | 1 | I |
| *Ehrlichia equi* isolate CAMAWI | AF172160 | Horse | USA | 1 | I |
| *Ehrlichia* sp. 'HGE agent' isolate CAHU-HGE2 | AF172159 | Human | USA | 1 | I |
| *Ehrlichia equi* isolate CASOLJ | AF172158 | Horse | USA | 1 | I |
| *Anaplasma phagocytophilum* strain GACTR12 | DQ088133 | *Sylvilagus floridanus* | USA | 1 | I |
| *Anaplasma phagocytophilum* isolate 151 | EU157921 | *Capreolus capreolus* | Poland | 1 | I |
| *Anaplasma phagocytophilum* strain ST-156 | DQ779567 | *Cervus elaphus* | Poland | 1 | I |
| *Anaplasma phagocytophilum* isolate 9B13 | KC800986 | *Alces alces* | Sweden | 1 | I |
| *Anaplasma phagocytophilum* isolate Nf_DU1_HW | JF494841 | *Neotoma fuscipes* | USA | 1 | I |
| *Anaplasma phagocytophilum* isolate Nf_1603_HV | JF494836 | *Neotoma fuscipes* | USA | 1 | I |
| *Anaplasma phagocytophilum* isolate Nf_1629_HC | JF494835 | *Neotoma fuscipes* | USA | 1 | I |
| *Anaplasma phagocytophilum* isolate Nf_1619_HC | JF494834 | *Neotoma fuscipes* | USA | 1 | I |
| *Anaplasma phagocytophilum* isolate Dog_CA | JF494833 | Dog | USA | 1 | I |
| *Ehrlichia* sp. 'HGE agent' | U72628 | Human | USA | 1 | I |
| *Anaplasma phagocytophilum* str. JM | CP006617 | Human | USA | 1 | I |
| *Anaplasma phagocytophilum* str. Dog2 | CP006618 | Dog | USA | 1 | I |
| *Anaplasma phagocytophilum* strain Webster | EU860090 | Ruminants | France | 1 | I |
| *Anaplasma phagocytophilum* isolate GV348 | MK341070 | *Ixodes ricinus* | Slovakia | 1 | I |
| *Anaplasma phagocytophilum* isolate 14DRS | KR092132 | *Sus scrofa* | Slovakia | 1 | I |
| *Anaplasma phagocytophilum* isolate J | KF312361 | *Ixodes ricinus* | Poland | 1 | I |
| *Anaplasma phagocytophilum* isolate G | KF312358 | *Ixodes ricinus* | Poland | 1 | I |
| *Anaplasma phagocytophilum* isolate F | KF312360 | *Ixodes ricinus* | Poland | 1 | I |
| *Anaplasma phagocytophilum* isolate E | KF312357 | *Ixodes ricinus* | Poland | 1 | I |
| *Anaplasma phagocytophilum* isolate I | KF312359 | *Ixodes ricinus* | Poland | 1 | I |
| *Anaplasma phagocytophilum* isolate H | KF312355 | *Ixodes ricinus* | Poland | 1 | I |
| *Anaplasma phagocytophilum* isolate Z15 | MW762533 | *Lepus europaeus* | Czech Republic | 1 | I |
| *Anaplasma phagocytophilum* isolate 16Pl | MG670108 | *Procyon lotor* | Poland | 1 | I |
| *Anaplasma phagocytophilum* strain ItalyIRH01241 | KF031388 | *Ixodes ricinus* | Italy | 1 | I |
| *Anaplasma phagocytophilum* isolate 2916 | EU860087 | Cow | France | 1 | I |
| *Anaplasma phagocytophilum* isolate 811 | AF478551 | *Capreolus capreolus* | Slovenia | 3 | II |
| *Anaplasma phagocytophilum* isolate 470 | AF478559 | *Capreolus capreolus* | Slovenia | 3 | II |
| *Anaplasma phagocytophilum* strain rod-1427 | KM215256 | *Capreolus capreolus* | Slovenia | 3 | II |
| *Anaplasma phagocytophilum* isolate D21 | AY281816 | *Ixodes ricinus* | Germany | 3 | II |
| *Anaplasma phagocytophilum* isolate 805 | AF478555 | *Capreolus capreolus* | Slovenia | 3 | II |
| *Anaplasma phagocytophilum* strain rod-1693 | KM215255 | *Capreolus capreolus* | Slovenia | 3 | II |
| *Anaplasma phagocytophilum* strain tick-EU136 | KM215250 | *Ixodes ricinus* | Slovenia | 3 | II |
| *Anaplasma phagocytophilum* isolate G26 | AY281820 | *Ixodes ricinus* | Germany | 3 | II |
| *Anaplasma phagocytophilum* isolate 794 | AF478556 | *Capreolus capreolus* | Slovenia | 3 | II |
| *Anaplasma phagocytophilum* strain tick-EU260 | KM215249 | *Ixodes ricinus* | Slovenia | 3 | II |
| *Anaplasma phagocytophilum* isolate A4 GroEL | AY220469 | *Capreolus capreolus* | Austria | 3 | II |
| *Anaplasma phagocytophilum* strain rod-1424 | KM215259 | *Capreolus capreolus* | Slovenia | 3 | II |
| *Anaplasma phagocytophilum* strain rod-1694 | KM215258 | *Capreolus capreolus* | Slovenia | 3 | II |
| *Anaplasma phagocytophilum* isolate 806 | AF478554 | *Capreolus capreolus* | Slovenia | 3 | II |
| *Anaplasma phagocytophilum* isolate A6 | AY220470 | *Ixodes ricinus* | Austria | 3 | II |
| *Anaplasma phagocytophilum* strain tick-EU322 | KM215247 | *Ixodes ricinus* | Slovenia | 3 | II |
| *Ehrlichia phagocytophila* #56 | AF383225 | *Capreolus capreolus* | Switzerland | 3 | II |
| *Anaplasma phagocytophilum* strain rod-1429 | KM215257 | *Capreolus capreolus* | Slovenia | 3 | II |
| *Anaplasma phagocytophilum* isolate 478 | AF478564 | *Capreolus capreolus* | Slovenia | 3 | II |
| *Anaplasma phagocytophilum* strain tick-EU329 | KM215248 | *Ixodes ricinus* | Slovenia | 3 | II |
| *Anaplasma phagocytophilum* strain rod-1691 | KM215254 | *Capreolus capreolus* | Slovenia | 3 | II |
| *Anaplasma phagocytophilum* isolate I63 | AY281825 | *Ixodes ricinus* | Germany | 3 | II |
| *Anaplasma phagocytophilum* strain rod-1686 | KM215253 | *Capreolus capreolus* | Slovenia | 3 | II |
| *Anaplasma phagocytophilum* isolate 47 | JN005747 | *Capreolus capreolus* | Poland | 3 | II |
| *Anaplasma phagocytophilum* isolate 09 | JN005743 | *Capreolus capreolus* | Poland | 3 | II |
| *Anaplasma phagocytophilum* strain Italy59_3 | KF031400 | *Ixodes ricinus* | Italy | 3 | II |
| *Anaplasma phagocytophilum* strain ItalyIRH018611 | KF031394 | *Ixodes ricinus* | Italy | 3 | II |
| *Anaplasma phagocytophilum* strain ItalyIRH012211 | KF031392 | *Ixodes ricinus* | Italy | 3 | II |
| *Anaplasma phagocytophilum* strain Italy25 | KF031382 | *Ixodes ricinus* | Italy | 3 | II |
| *Anaplasma phagocytophilum* strain Italy21b | KF031380 | *Ixodes ricinus* | Italy | 3 | II |
| *Anaplasma phagocytophilum* strain ST-128 | DQ779568 | *Capreolus capreolus* | Poland | 3 | II |
| *Anaplasma phagocytophilum* isolate S40 GroEL | KC800984 | *Alces alces* | Sweden | 3 | II |
| *Anaplasma phagocytophilum* isolate Italy737 | EU552920 | *Ixodes ricinus* | Italy | 3 | II |
| *Anaplasma phagocytophilum* isolate Italy738 | EU552918 | *Ixodes ricinus* | Italy | 3 | II |
| *Anaplasma phagocytophilum* isolate K | KF312356 | *Ixodes ricinus* | Poland | 3 | II |
| *Anaplasma phagocytophilum* isolate fricoe | JX082324 | *Ixodes ricinus* | Switzerland | 3 | II |
| *Anaplasma phagocytophilum* isolate erirub | JX082325 | *Ixodes ricinus* | Switzerland | 3 | II |
| Uncultured *Anaplasma* sp. clone Ip11-2 | JQ622144 | *Ixodes persulcatus* | Japan | 4 | II |
| *Anaplasma phagocytophilum* isolate Kh-395_Ip | MN989865 | *Ixodes persulcatus* | Russia | 4 | II |
| *Anaplasma phagocytophilum* isolate Kh-434_Ip | MN989863 | *Ixodes persulcatus* | Russia | 4 | II |
| *Anaplasma phagocytophilum* isolate Irk-Ip776 | HM366573 | *Ixodes persulcatus* | Russia | 4 | II |
| *Anaplasma phagocytophilum* isolate Irk-Ip820 | HM366574 | *Ixodes persulcatus* | Russia | 4 | II |
| *Anaplasma phagocytophilum* isolate Kh-chipmunk177 | HQ630619 | *Tamias sibiricus* | Russia | 4 | II |
| *Anaplasma phagocytophilum* isolate Irk-Ip662 | HM366572 | *Ixodes persulcatus* | Russia | 4 | II |
| *Anaplasma phagocytophilum* isolate Kh-Ip7 | HM366575 | *Ixodes persulcatus* | Russia | 4 | II |
| *Anaplasma phagocytophilum* isolate Kh-Ip80 | HM366576 | *Ixodes persulcatus* | Russia | 4 | II |
| *Anaplasma phagocytophilum* isolate Kh-Ip144 | HM366577 | *Ixodes persulcatus* | Russia | 4 | II |
| *Anaplasma phagocytophilum* isolate Kh-868_Tsib | MN989862 | *Tamias sibiricus* | Russia | 4 | II |
| *Anaplasma phagocytophilum* isolate RUS/Nov14-1682-Ipr/Ipv | MG182154 | *Ixodes persulcatus* | Russia | 4 | II |
| *Anaplasma phagocytophilum* isolate RUS/Nov14-1768-Ipv | KX980043 | *Ixodes pavlovskyi* | Russia | 4 | II |
| *Anaplasma phagocytophilum* isolate Nov-chipmunk1322 | HQ630618 | *Tamias sibiricus* | Russia | 4 | II |
| *Anaplasma phagocytophilum* isolate Sv-Ip854 | HM366567 | *Ixodes persulcatus* | Russia | 4 | II |
| *Anaplasma phagocytophilum* isolate Nov-Ip364 | HM366569 | *Ixodes persulcatus* | Russia | 4 | II |
| *Anaplasma phagocytophilum* isolate Omsk-373_Ip | MN701641 | *Ixodes persulcatus* | Russia | 4 | II |
| *Anaplasma phagocytophilum* clone Omsk-vole83 | KC583433 | *Myodes glareolus* | Russia | 4 | II |
| *Anaplasma phagocytophilum* isolate Nov-Ip355 | HM366568 | *Ixodes persulcatus* | Russia | 4 | II |
| *Anaplasma phagocytophilum* isolate Kh-Ip160 | HM366578 | *Ixodes persulcatus* | Russia | 4 | II |
| *Anaplasma phagocytophilum* strain AAIK2 GroES | KT220191 | *Apodemus agrarius* | South Korea | 4 | II |
| *Anaplasma phagocytophilum* strain AAIK3 | KT220192 | *Apodemus agrarius* | South Korea | 4 | II |
| *Anaplasma phagocytophilum* strain AAIK1 | KT192430 | *Apodemus agrarius* | South Korea | 4 | II |
| *Anaplasma phagocytophilum* isolate Hongdo-11-1 | JX219474 | *Ixodes nipponensis* | South Korea | 4 | II |
| *Anaplasma phagocytophilum* clone KWDTAPg | JQ086319 | *Haemaphysalis flava* | South Korea | 4 | II |
| *Anaplasma phagocytophilum* isolate KWDAPg | HM752098 | *Hydropotes inermis* | South Korea | 4 | II |
| *Anaplasma phagocytophilum* Yeyasu | LC334016 | Dog | Japan | 4 | II |
| *Anaplasma phagocytophilum* isolate gw1 | KJ677107 | Human | South Korea | 4 | II |
| *Anaplasma phagocytophilum* isolate D-SE-63 | KU519286 | Dog | South Korea | 4 | II |
| *Anaplasma phagocytophilum* isolate S-DD-20 | KU519284 | Cat | South Korea | 4 | II |
| *Anaplasma phagocytophilum* strain N6Bel | JX133177 | *Ixodes ricinus* | Switzerland | 3 | II |
| *Anaplasma phagocytophilum* strain ItalyIRH017411 | KF031393 | *Ixodes ricinus* | Italy | 7 | IV |
| *Anaplasma phagocytophilum* isolate turmer | JX082323 | *Ixodes ricinus* | Switzerland | 7 | IV |
| ***Anaplasma phagocytophilum* strain Patagonia 5P** | **OP585578** | ***Pudu puda*** | **Chile** | **8** | **V** |
| ***Anaplasma phagocytophilum* strain Patagonia 7P** | **OP585579** | ***Pudu puda*** | **Chile** | **8** | **V** |
| ***Anaplasma phagocytophilum* strain Patagonia IS8** | **OP585581** | ***Ixodes stilesi*** | **Chile** | **8** | **V** |
| ***Anaplasma phagocytophilum* strain Patagonia IS17** | **OP585582** | ***Ixodes stilesi*** | **Chile** | **8** | **V** |
| ***Anaplasma phagocytophilum* strain Patagonia IS21** | **OP585585** | ***Ixodes stilesi*** | **Chile** | **8** | **V** |
| ***Anaplasma phagocytophilum* strain Patagonia 36S** | **OP585589** | ***Pudu puda*** | **Chile** | **8** | **V** |
| ***Anaplasma phagocytophilum* strain Patagonia 24P** | **OP585590** | ***Pudu puda*** | **Chile** | **8** | **V** |
| ***Anaplasma phagocytophilum* strain Patagonia IS25** | **OP585588** | ***Ixodes stilesi*** | **Chile** | **8** | **V** |
| ***Anaplasma phagocytophilum* strain Patagonia 8P** | **OP585580** | ***Pudu puda*** | **Chile** | **8** | **V** |
| ***Anaplasma phagocytophilum* strain Patagonia IS20** | **OP585586** | ***Ixodes stilesi*** | **Chile** | **8** | **V** |
| ***Anaplasma phagocytophilum* strain Patagonia IS22** | **OP585587** | ***Ixodes stilesi*** | **Chile** | **8** | **V** |
| ***Anaplasma phagocytophilum* strain Patagonia IS18** | **OP585583** | ***Ixodes stilesi*** | **Chile** | **8** | **V** |
| ***Anaplasma phagocytophilum* strain Patagonia IS19** | **OP585584** | ***Ixodes stilesi*** | **Chile** | **8** | **V** |
| *Anaplasma phagocytophilum* | AY279085 | Goat | Albania | Unassigned | Unassigned |
| *Anaplasma phagocytophilum* isolate 77HNIRQ | KF383240 | *Ixodes ricinus* | Slovakia | Unassigned | Unassigned |
| *Anaplasma phagocytophilum* isolate 5NBZIRQ | KF383238 | *Ixodes ricinus* | Slovakia | Unassigned | Unassigned |
| *Anaplasma phagocytophilum* isolate 42 | EU157920 | *Capreolus capreolus* | Poland | Unassigned | Unassigned |
| *Anaplasma platys* strain RP | EU516386 |  |  | Not applicable | Not applicable |
| *Anaplasma platys* strain WHBMXZ-126 | KX987394 | *Boophilus microplus* | China | Not applicable | Not applicable |
| *Anaplasma odocoilei* strain UMUM76 | JX876642 | *Odocoileus virginianus* |  | Not applicable | Not applicable |
| *Anaplasma marginale* isolate CNP_976_2 | KY305561 | *Syncerus caffer* | South Africa | Not applicable | Not applicable |
| *Anaplasma marginale* isolate AEP_1007_3 | KY305562 | *Syncerus caffer* | South Africa | Not applicable | Not applicable |
| *Anaplasma centrale* isolate Ac1_Ug_Ktd_2013_KR5_30h | KY523000 | Cattle | Uganda | Not applicable | Not applicable |
| *Anaplasma ovis* isolate OVI | AF441131 |  | South Africa | Not applicable | Not applicable |
| *Anaplasma ovis* isolate 76 | FJ460441 |  | Cyprus | Not applicable | Not applicable |
| *Anaplasma capra* strain HLJ-14 | KM206275 | Human | China | Not applicable | Not applicable |
| *Anaplasma capra* isolate Hstaji200 | MZ222248 | *Equus hemionus onager* | Iran | Not applicable | Not applicable |
| *Ehrlichia ruminantium* strain Kumm2 | DQ647013 |  | South Africa | Not applicable | Not applicable |
